# Nocardamine mitigates cellular dysfunction induced by oxidative stress in periodontal ligament stem cells

**DOI:** 10.1186/s13287-024-03812-2

**Published:** 2024-08-07

**Authors:** Hai-Peng He, Mei-Zhen Zhao, Wei-Hua Jiao, Zhi-Qiang Liu, Xian-Hai Zeng, Quan-Li Li, Tian-Yong Hu, Bao-Hui Cheng

**Affiliations:** 1 Department of Dentistry, Shenzhen Longgang Otolaryngology hospital & Shenzhen Otolaryngology Research Institute, Shenzhen, 518172 China; 2 Research Center for Marine Drugs, State Key Laboratory of Microbial Metabolism, Department of Pharmacy, Ren Ji Hospital, School of Medicine, Shanghai Jiao Tong University, Shanghai, 200127 China

**Keywords:** Periodontal destruction, Periodontal ligament stem cells, Oxidative stress, Nocardamine, Osteogenic differentiation

## Abstract

**Background:**

The role of periodontal ligament stem cells (PDLSCs) in repairing periodontal destruction is crucial, but their functions can be impaired by excessive oxidative stress (OS). Nocardamine (NOCA), a cyclic siderophore, has been shown to possess anti-cancer and anti-bacterial properties. This study aimed to investigate the protective mechanisms of NOCA against OS-induced cellular dysfunction in PDLSCs.

**Methods:**

The cytotoxicity of NOCA on PDLSCs was assessed using a CCK-8 assay. PDLSCs were then treated with hydrogen peroxide (H_2_O_2_) to induce OS. ROS levels, cell viability, and antioxidant factor expression were analyzed using relevant kits after treatment. Small molecule inhibitors U0126 and XAV-939 were employed to block ERK signaling and Wnt pathways respectively. Osteogenic differentiation was assessed using alkaline phosphatase (ALP) activity staining and Alizarin Red S (ARS) staining of mineralized nodules. Expression levels of osteogenic gene markers and ERK pathway were determined via real-time quantitative polymerase chain reaction (RT-qPCR) or western blot (WB) analysis. β-catenin nuclear localization was examined by western blotting and confocal microscopy.

**Results:**

NOCA exhibited no significant cytotoxicity at concentrations below 20 µM and effectively inhibited H_2_O_2_-induced OS in PDLSCs. NOCA also restored ALP activity, mineralized nodule formation, and the expression of osteogenic markers in H_2_O_2_-stimulated PDLSCs. Mechanistically, NOCA increased p-ERK level and promoted β-catenin translocation into the nucleus; however, blocking ERK pathway disrupted the osteogenic protection provided by NOCA and impaired its ability to induce β-catenin nuclear translocation under OS conditions in PDLSCs.

**Conclusions:**

NOCA protected PDLSCs against H_2_O_2_-induced OS and effectively restored impaired osteogenic differentiation in PDLSCs by modulating the ERK/Wnt signaling pathway.

**Supplementary Information:**

The online version contains supplementary material available at 10.1186/s13287-024-03812-2.

## Introduction

Periodontal tissue destruction, encompassing the periodontal ligament, alveolar bone, and gingiva, remains a significant health concern [1, 2]. Excessive oxidative stress (OS) is a key driver of this destruction, implicated in major risk factors like chronic inflammation in periodontitis, hyperglycemia in diabetes, and mitochondrial dysfunction in senescence [1, 2]. During OS progression, reactive oxygen species (ROS) skyrocket, disrupting the delicate balance within the endogenous antioxidant system. This OS onslaught impairs vital functions of periodontal ligament stem cells (PDLSCs), vital players in maintaining and repairing periodontal tissue [3, 4]. Consequently, mitigating this excessive OS response emerges as a crucial strategy to restore PDLSC function and halt periodontal tissue destruction.

Marine-derived natural products offer a treasure trove of unexplored bioactivities with potential therapeutic application [5, 6]. Nocardamine (NOCA), also called desferrioxamine E, is a group of siderophores that act as ferric ion chelating agents excreted by microorganisms during iron deficiency conditions [7]. NOCA is synthesized by various microorganisms and has been successfully reported with anti-cancer and anti-pathogenic microorganism properties [8, 9]. However, its antioxidative capabilities and underlying mechanisms in PDLSCs remain largely uncharted.

In this study, we obtained NOCA from a *Dysidea* sp. Marine Sponge and demonstrated its ability to repair cellular dysfunction mediated by OS in PDLSCs. However, the detailed antioxidative effects and related molecular mechanisms of NOCA have not been studied yet. Therefore, our aim was to investigate the protective mechanism of NOCA on PDLSC function under OS conditions to provide an experimental basis for clinical treatment of periodontal destruction.

## Materials and methods

### Reagents

NOCA is obtained from a *Dysidea* sp. Marine Sponge. Collagenase type I, dispase, MEM Alpha (aMEM), fetal bovine serum (FBS), and penicillin/streptomycin were purchased from Gibco (Grand Island, NY, USA). β-Glycerophosphate, dexamethasone, L-ascorbic acid, indomethacin, 3-Isobutyl-1-methylxanthine, Alizarin Red S, Oil Red O, Alcian-blue staining solution and XAV-939 were purchased from Sigma-Aldrich (St Louis, MO). Cluster of differentiation 73 (CD73)/FITC, CD90/PerCP-Cy™5.5, and CD45/FITC were purchased from BD Pharmingen (San Diego, CA). Alkaline phosphatase color development kit, Dihydroethidium, ROS assay kit, Superoxide Dismutase (SOD) assay kit, Catalase (CAT) assay kit, Bovine Serum Albumin (BSA), β-catenin antibody, Lamin B1 antibody, DAPI staining solution, and U0126 were purchased from Beyotime (Shanghai, China). Primary antibodies against SIRT1, COL1A1, phospho-Akt (p-Akt), Akt, p-ERK, ERK and GAPDH were purchased from Cell Signaling Technology (Danvers, MA, USA). RUNX2 antibody were purchased from Santa Cruz Biotechnology (USA). In addition, other reagents are explicitly stated.

### Cell culture

PDLSCs were isolated from molar teeth obtained with informed consent from three healthy human donors (18–24 years old), following the previously described protocol [10, 11]. Briefly, freshly harvested periodontal ligament tissue was cleaned, minced, and incubated in a solution containing 3 mg/mL collagenase type I and 4 mg/mL dispase at 37 °C for 1 h. The cell-containing solution was passed through a 70-µm strainer (BD Biosciences, Franklin Lakes, NJ), and the obtained single-cell suspension was cultured in 75 cm^2^ cell culture flasks using standard medium consisting of aMEM supplemented with 10% (v/v) FBS and 1% (v/v) penicillin/streptomycin under 5% CO_2_ at 37 °C. Upon reaching approximately 80% confluence, the cells were subcultured. For all experiments described below, PDLSCs from passages 3 to 5 were utilized.

### Characterization of PDLSCs

As per the literature previously reported [11, 12], isolated PDLSCs were characterized for their mesenchymal stem cell (MSC) markers before experimentation. This study protocol was approved by the Ethics Committee of Longgang Otolaryngology Hospital, Institute of Otolaryngology, and Shenzhen Key Laboratory of Otolaryngology (2021 − 0159). Flow cytometry confirmed their expression of MSC markers CD73 and CD90 while lacking the hematopoietic marker CD45. To validate their multilineage differentiation potential, PDLSCs were cultured in osteogenic, adipogenic, and chondrogenic induction media. Osteogenic differentiation was induced in standard medium supplemented with 10 mM β-Glycerophosphate, 100 nM dexamethasone, and 50 µg/mL L-ascorbic acid. Adipogenic differentiation was induced in standard medium supplemented with 1 μM dexamethasone, 100 µM indomethacin, 500 µM 3-Isobutyl-1-methylxanthine, and 50 µg/mL L-ascorbic acid. Chondrogenic differentiation was induced using the Chondrogenic Differentiation Bullet Kit (Lonza, Walkersville, MD, USA, catalog number PT-3003 and PT-4124) according to the manufacturer’s instructions. The medium was changed twice weekly. After three weeks of induction, the cells were fixed with 4% paraformaldehyde and stained with Alizarin Red S (ARS) for osteogenesis, Oil Red O (ORO) for adipogenesis, and Alcian blue (AB) for chondrogenesis.

### Cell viability assay

PDLSCs or other cell lines were seeded at a density of 1 × 10^4^ cells in 100 µL of standard medium per well in 96-well plates and incubated for 24 h. Subsequently, the cells were exposed to various concentrations of NOCA (ranging from 0 to 320 µM) in fresh complete α-MEM for an additional 24 h. For analysis of oxidative stress protection, cells were pretreated for 4 hours with either 5 µM or 10 µM NOCA or 10 µM Quercetin (Guangzhou Institute for Drug Control, Guangzhou, China) in standard medium before stimulation with 150 µM H_2_O_2_ (Sigma-Aldrich, St. Louis, MO) for 24 h in standard medium. Cell viability was then assessed using the CCK-8 assay. Briefly, 10 µl of CCK-8 solution (Dojindo Molecular Technologies, Inc., Kumamoto, Japan) was added to each well and incubated for 2 h. Finally, absorbance measurements were obtained at 450 nm using the SpectraMax Paradigm Multi-Mode Microplate Reader (Molecular Devices, San Jose, CA, USA).

### Intracellular ROS and antioxidation assay

Intracellular ROS levels were assessed using both 5 µM Dihydroethidium (DHE) and 10 µM 2’,7’-dichlorofluorescein diacetate (DCFH). Briefly, cells were incubated with the respective dye at 37 °C for 20 min in the dark and then analyzed. DHE fluorescence was visualized using a fluorescence microscope (Leica DMI3000 B). DCFH fluorescence was measured by flow cytometry (BD FACSAriaTM II). To explore potential mechanisms of ROS modulation, Superoxide Dismutase (SOD) activity and Catalase (CAT) activity were determined using corresponding assay kits (Beyotime) according to the manufacturer’s instructions.

### Osteogenic differentiation assay

PDLSCs were cultured in osteogenic medium supplemented with 10 mM β-glycerophosphate, 100 nM dexamethasone, and 50 µg/mL L-ascorbic acid in standard medium. After 7 days and 14 days of culture, cells were fixed with 4% paraformaldehyde and assessed for osteogenic differentiation using Alkaline phosphatase (ALP) staining and Alizarin Red S (ARS) staining, respectively. Images were acquired using a Sharp Corporation scanner and microscope. To further confirm osteogenic potential, the expression levels of Runt-related transcription factor 2 (RUNX2), ALP, and Collagen type I alpha 1 chain (COL1A1) were analyzed by real-time quantitative polymerase chain reaction (RT-qPCR) using a 7500 Real-Time PCR System (Applied Biosystems, Foster City, CA) and Western blot (WB) analysis using specific antibodies against RUNX2, ALP, and COL1A1.

### RT-qPCR assay

Total RNA was extracted from PDLSC lysate using the Trizol reagent (Invitrogen, Carlsbad, CA). cDNA synthesis (1 µg) was then performed using the PrimeScript TM RT reagent Kit with gDNA Eraser (TaKaRa, Tokyo, Japan) following the manufacturer’s instructions. RT-qPCR was conducted using the SYBR^®^ Premix Ex Taq TM II kit (TaKaRa) in a total reaction volume of 20 µL on a 7500 Real-Time PCR System. The specific primer sequences used for target gene amplification are listed in Table 1.

**Table 1:** Primer sequences

Primer	Sequences (5’-3’)
SIRT1	Forward TAGCCTTGTCAGATAAGGAAGGA
	Reverse ACAGCTTCACAGTCAACTTTGT
RUNX2	Forward CTCCAACCCACGAATGCACTA
	Reverse GTGAGTGGGTGGCGGACATG
ALP	Forward AGCCCTTCACTGCCATCCTGT
	Reverse ATTCTCTCGTTCACCGCCAC
COL1A1	Forward GAGGGCCAAGACGAAGACATC
	Reverse CAGATCACGTCATCGCACAAC
GAPDH	Forward GGCATGGACTGTGGTCATGAG
	Reverse TGCACCACCAACTGCTTAGC

### WB assay

Protein was extracted from cultured PDLSCs using either RIPA Lysis Buffer (50 mM Tris-HCl, pH 7.4, 150 mM NaCl, 1% Nonidet P-40, 0.5% sodium deoxycholate, 0.1% SDS, and protease inhibitor cocktail) or the Nucleoprotein Extraction Kit (Sangon Biotech, Shanghai, China) and quantified by the BCA protein assay kit (Thermo Scientific, IL) to determine protein concentration. Subsequently, 40 µg of protein samples were separated by sodium dodecyl sulfate polyacrylamide gel electrophoresis and transferred onto PVDF membranes. The membranes were then blocked with either 5% non-fat milk or 5% BSA for 1 h at room temperature, followed by overnight incubation at 4 °C with primary antibodies targeting specific proteins of interest. Afterwards, the membranes were incubated with a horseradish peroxidase (HRP)-conjugated secondary antibody at room temperature for 1 h, and finally visualized using PierceTM ECL Western Blotting Substrate (Thermo Scientific). The detection was performed using a Bio-Rad ChemiDocMP System, while quantification of the WB results was conducted utilizing ImageJ software.

### Immunofluorescent staining

The cells were fixed with 4% paraformaldehyde for 30 min, permeabilized with 0.1% Triton X-100 for 15 min, and blocked with 2% BSA for 1 h. Afterward, they were incubated with β-catenin antibody at a dilution of 1:100 (or specify according to your experiment) overnight at 4 °C. The next day, the cells were incubated with a fluorescently conjugated secondary antibody (Abcam, Cambridge, MA, USA) for 1 h at room temperature. Nuclear staining was performed with DAPI staining solution for 10 min. Images were acquired using Laser Scanning Confocal Microscopy (Leica TCS SP5 II).

### Statistical analysis

The statistical analysis results were reported as the mean ± standard error of the mean. Differences between each data set were assessed using the Student t test. Statistical significance was defined as P values < 0.05.

## Results

### Characterization of PDLSCs

**Fig. 1:**
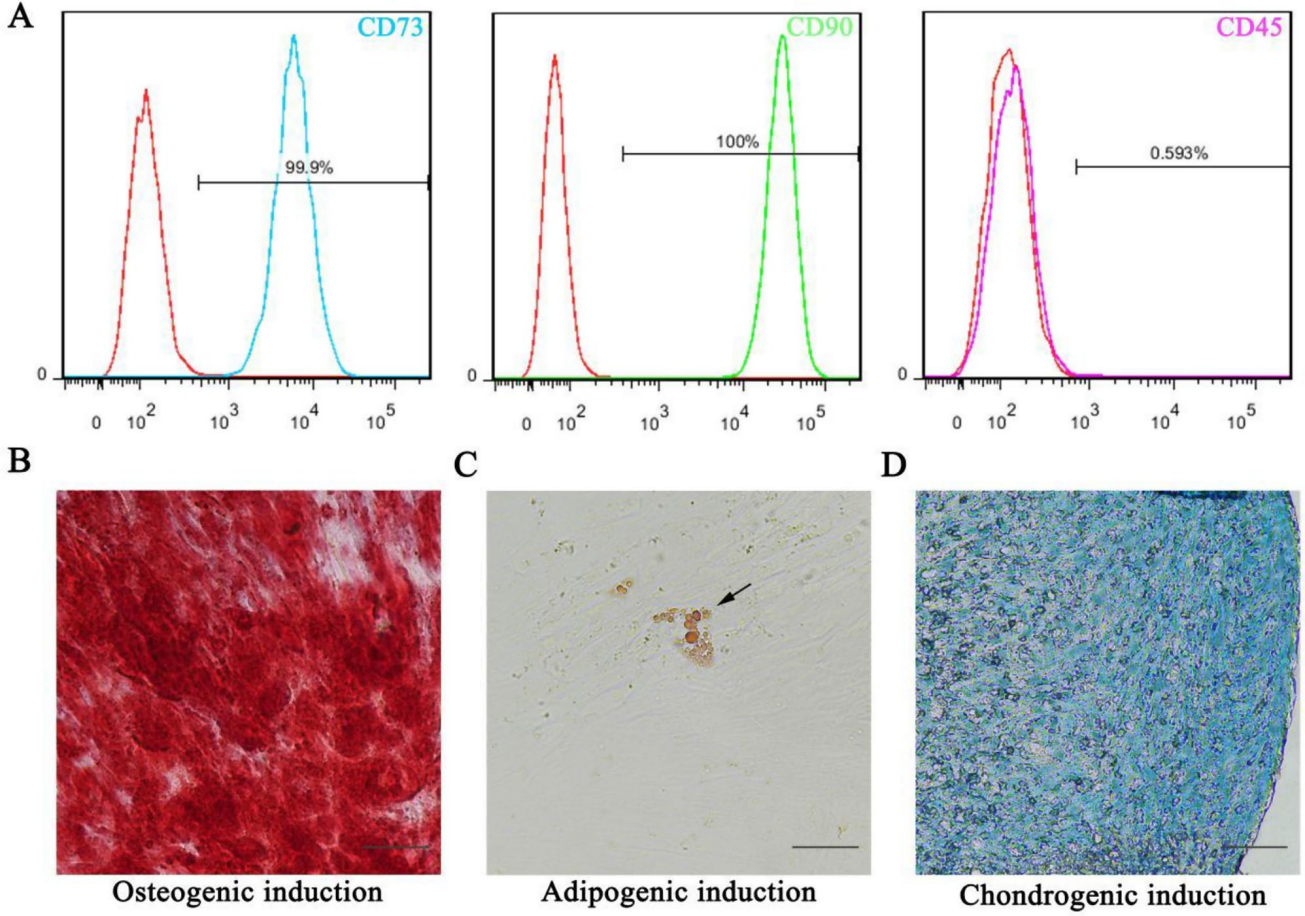
Characterization of PDLSCs. (**A**) Flow cytometry analysis was performed to evaluate the expression of MSCs-related phenotypic markers, including CD73, CD90, and CD45. (**B**) Mineralized nodules were stained using ARS staining solution to assess osteogenic differentiation after 3 weeks of induction. (**C**) Lipid droplets were visualized by ORO staining to examine adipogenic differentiation after 3 weeks of induction. (**D**) Chondrocyte-like cells were identified through AB staining to investigate chondrogenic differentiation after 3 weeks of induction. (Scale bar = 50 μm)

Flow cytometry analysis confirmed the isolated cells as mesenchymal stem cells, with 99.9% and 100% of cells positive for CD73 and CD90, respectively, and no detectable CD45 expression (Fig. 1A). Further characterization revealed their multipotent differentiation potential. When cultured in osteogenic medium, the cells formed mineralized nodules stained positively with ARS (Fig. 1B), displaying morphology consistent with bone matrix deposits. Adipogenic differentiation resulted in abundant lipid droplet formation, as evidenced by ORO staining (Fig. 1C). Chondrogenic induction led to the development of chondrocyte-like cells exhibiting blue-green staining with AB (Fig. 1D). These findings collectively provide strong evidence that the isolated cells possess multipotent differentiation potential, confirming their characteristics consistent with those of MSCs.

### Effects of NOCA on cell viability

**Fig. 2:**
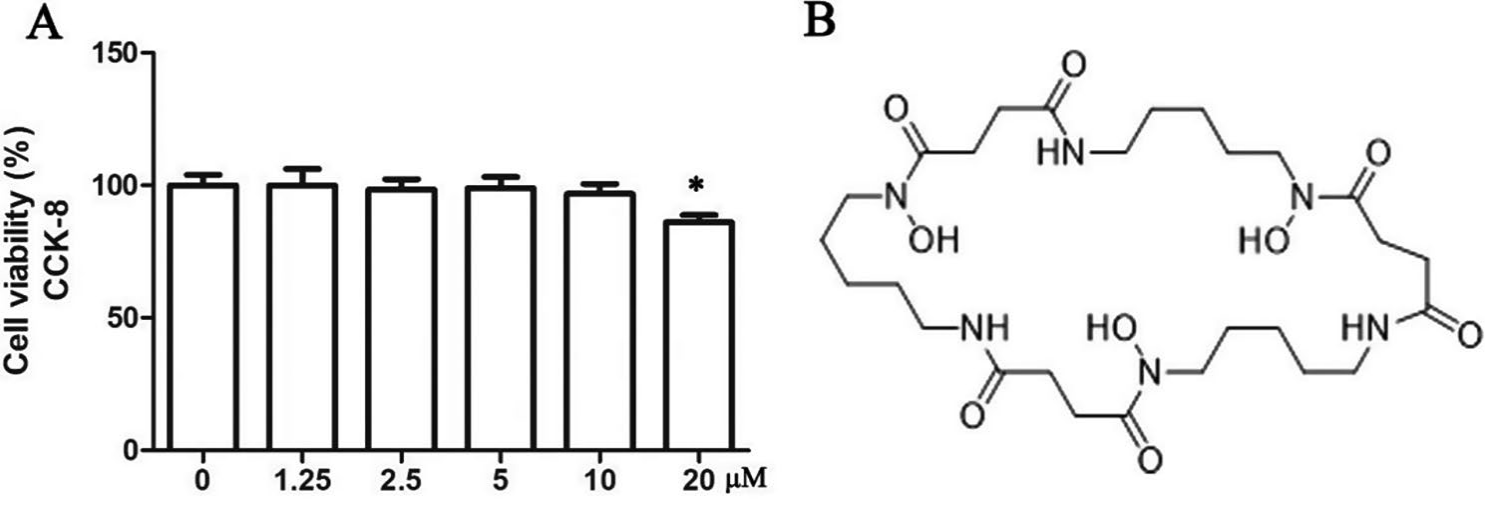
Following treatment with various concentrations of NOCA ranging from 0 µM to 20 µM for a duration of 24 h, cell viability was assessed using the CCK-8 assay. (**A**) Cytotoxic effects of NOCA on PDLSCs. (**B**) Chemical structure of NOCA. *P < 0.05 vs. NOCA 0 µM treated group, *n* = 3

To assess the cytotoxicity of NOCA, PDLSCs were cultured with varying concentrations of NOCA for 24 h. The results indicated that NOCA did not significantly impact cell viability at levels below 20 µM (Fig. 2A). We further assessed the cytotoxicity of NOCA on various cell lines. The findings revealed that concentrations below 20 μm did not exert a significant inhibitory effect on human dental pulp stem cells (DPSCs), human gingival fibroblasts (HGFs) and MC3T3-E1 (a pre-osteoblast cell line)(Fig. S1). Therefore, subsequent experiments were conducted using concentrations lower than 20 µM.

### NOCA attenuated H_2_O_2_-induced OS in PDLSCs

**Fig. 3:**
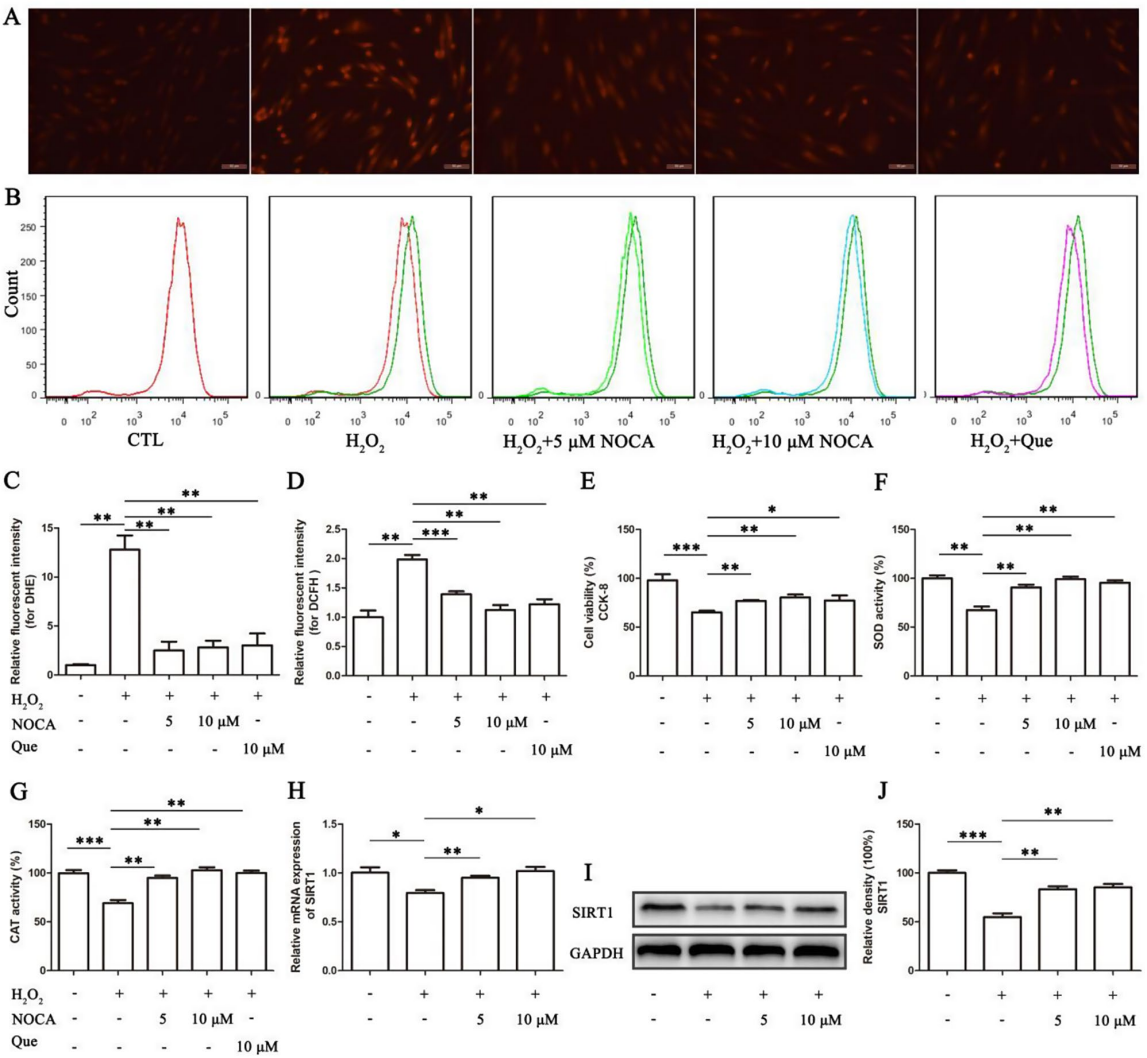
NOCA attenuated H_2_O_2_-induced OS in PDLSCs. PDLSCs were pretreated with NOCA or Que for 4 h, followed by stimulation with 150 µM H_2_O_2_ for 24 h. The cells were labeled with DHE or DCFH for 20 min. Standard medium served as a control group (CTL), and Que was chosen as a reference control. (**A**, **C**) ROS levels were visualized using fluorescence microscopy (Scale bar = 50 μm). (**B**, **D**) Flow cytometry was employed to detect ROS levels. (**E**) Cell viability assay, (**F**) SOD activity, and (**G**) CAT activity were measured using relevant kits. (**H**) SIRT1 mRNA expression was analyzed through RT-qPCR analysis. (**I**) SIRT1 protein expression evaluated via WB. (**J**) Relative band density of (**I**). *P < 0.05, **P < 0.01, ***P < 0.001 vs. NOCA 0 µM treated group, *n* = 3

To investigate whether NOCA attenuated H_2_O_2_-induced oxidative stress in PDLSCs, we first evaluated ROS production using the DCFH and DHE assay. Quercetin (Que), a widely recognized antioxidant, has been documented to mitigate the OS response of PDLSCs and restore their functionality [13, 14]. Therefore, we employed Que as a reference control in our study. As expected, H_2_O_2_ treatment significantly increased ROS levels compared to control (*p* < 0.05). Pretreatment with either NOCA (5 µM and 10 µM) or Que (10 µM) reduced ROS levels, with NOCA at 10 µM decreasing ROS by 40% compared to H_2_O_2_ alone (*p* < 0.05) (Fig. 3A-D). We then assessed cell viability by CCK-8 assay and observed a significant decrease in viability after H_2_O_2_ incubation (*p* < 0.05). This reduction was dose-dependently reversed by NOCA pretreatment, with viability at 10 µM NOCA similar to control levels (Fig. 3E). Similarly, H_2_O_2_ significantly decreased SOD activity and CAT concentration by more than 30% compared to the control group (*p* < 0.05). NOCA treatment again provided dose-dependent protection, with 10 µM NOCA restoring SOD and CAT activity to near control levels (*p* > 0.05) (Fig. 3F, G). Finally, to explore a potential mechanism of NOCA’s antioxidative effects, we analyzed the expression levels of Sirtuin 1 (SIRT1), a key transcription factor involved in antioxidant gene regulation [15]. Notably, H_2_O_2_ treatment significantly suppressed both SIRT1 mRNA expression by 20% and SIRT1 protein levels by 50% compared to the control group (*p* < 0.05). However, pretreatment with increasing doses of NOCA dose-dependently restored SIRT1 levels to at least 85% of the control group (*p* < 0.05), as measured by RT-qPCR and WB (Fig. 3H-J). Collectively, these findings suggest that NOCA’s ability to mitigate H_2_O_2_-induced OS in PDLSCs might be mediated, at least in part, through the upregulation of SIRT1 expression, leading to enhanced antioxidant activity and protection against cellular damage.

### NOCA attenuated OS-impaired osteogenic differentiation in PDLSCs

**Fig. 4:**
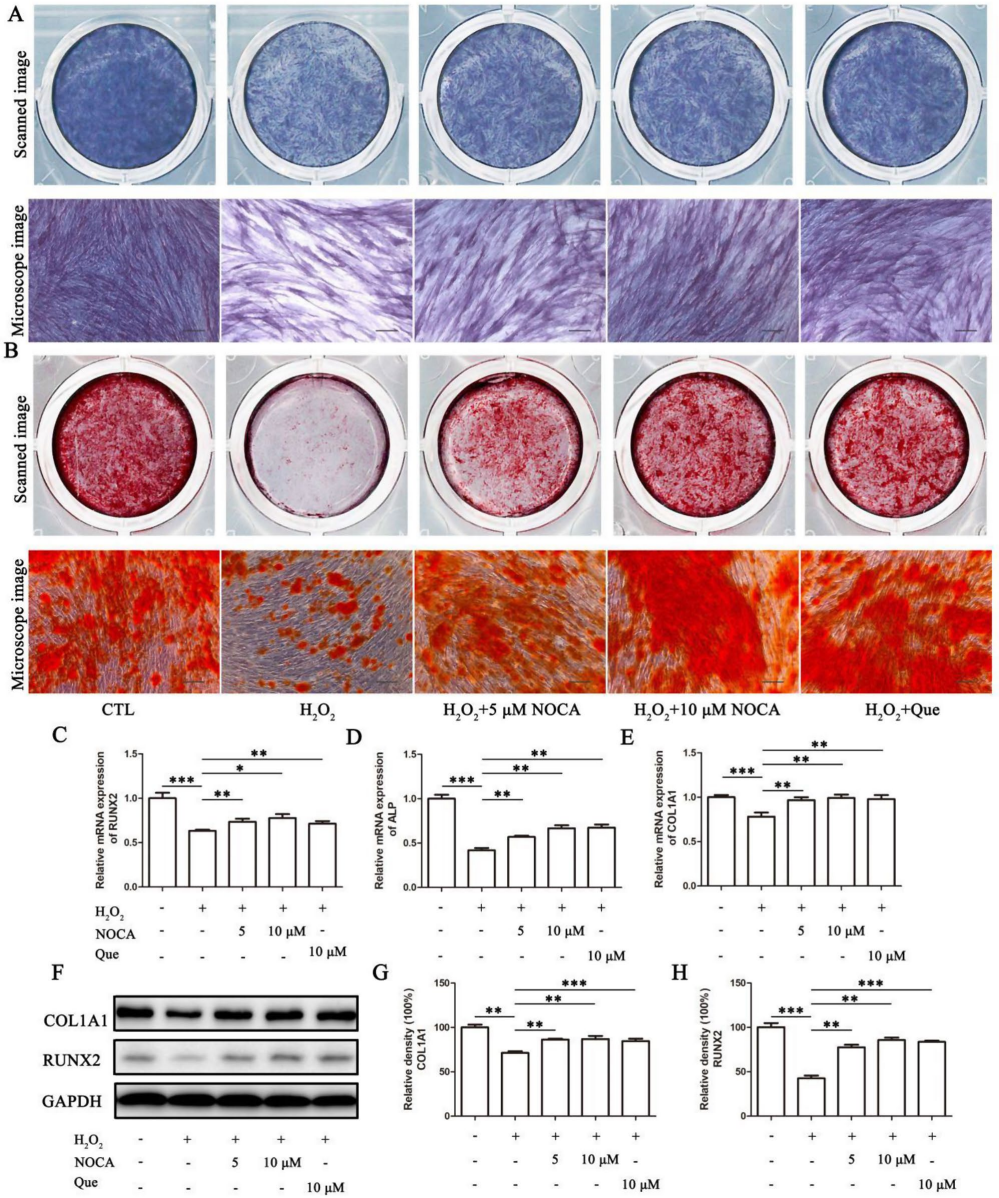
NOCA attenuated OS-impaired osteogenic differentiation in PDLSCs. After treatment with H_2_O_2_ and NOCA using a similar methodology as described above, the cells were subsequently induced in an osteogenic medium. Osteogenic media served as a control group (CTL), while Que was chosen as a reference control. (**A**) Images of ALP activity staining, and (**B**) ARS staining were acquired using a scanner and microscope (Scale bar = 100 μm). (**C**-**E**) mRNA expression levels of RUNX2, ALP, and COL1A1 were analyzed by RT-qPCR. (**F**) The protein expression levels of COL1A1 and RUNX2 were analyzed by WB. (**G**, **H**) Relative band density of (**F**). *P < 0.05, **P < 0.01, ***P < 0.001 vs. NOCA 0 µM treated group, *n* = 3

**Fig. 5:**
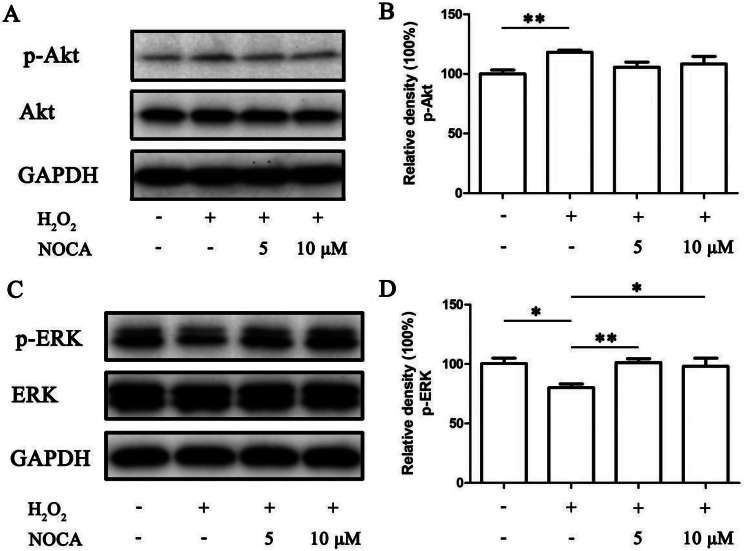
Effects of NOCA on the p-Akt and p-ERK in PDLSCs under OS. (**A**) The protein levels of p-Akt and Akt. (**B**) Relative band density of (**A**). (**C**) The protein levels of p-ERK and ERK. (**D**) Relative band density of (**C**). *P < 0.05, **P < 0.01 vs. NOCA 0 µM treated group, *n* = 3

To investigate whether NOCA could attenuate H_2_O_2_-induced impairment of osteogenic differentiation, PDLSCs were subjected to differentiation medium for 7 and 14 days following a 24-hour treatment with H_2_O_2_ and/or NOCA/Que. Osteogenic differentiation was assessed by ALP activity, calcium nodule formation, and the expression of key osteogenic markers. As expected, H_2_O_2_ treatment significantly reduced ALP activity, calcium nodule formation, and the expression of all three osteogenic markers (*p* < 0.05). Notably, both NOCA and Que improved ALP activity, with 10 µM NOCA to near control levels. Similarly, NOCA/Que administration partially restored calcium nodule formation and increased the expression of osteogenic markers, suggesting their protective potential against H_2_O_2_-induced damage (Fig. 4). To elucidate the possible mechanisms underlying NOCA’s protective effects, we further examined the activation of two well-known antioxidant pathways - Akt and ERK signaling pathways [16, 17]. Our findings revealed that after H_2_O_2_ treatment, p-Akt levels were increased by less than 20% compared to control (*p* < 0.05), while p-ERK levels were significantly decreased (*p* < 0.05). Notably, pretreatment with NOCA did not significantly alter p-Akt levels but partially restored p-ERK levels to near control (*p* < 0.05) during osteogenic differentiation in the presence of H_2_O_2_. These results suggest that NOCA’s ability to mitigate H_2_O_2_-induced impairment of PDLSCs’ osteogenic potential might be independently mediated through the ERK pathway, while bypassing the Akt signaling cascade (Fig. 5).

### The ERK/Wnt pathway mediated the protective effects of NOCA on OS-impaired osteogenic differentiation in PDLSCs

**Fig. 6:**
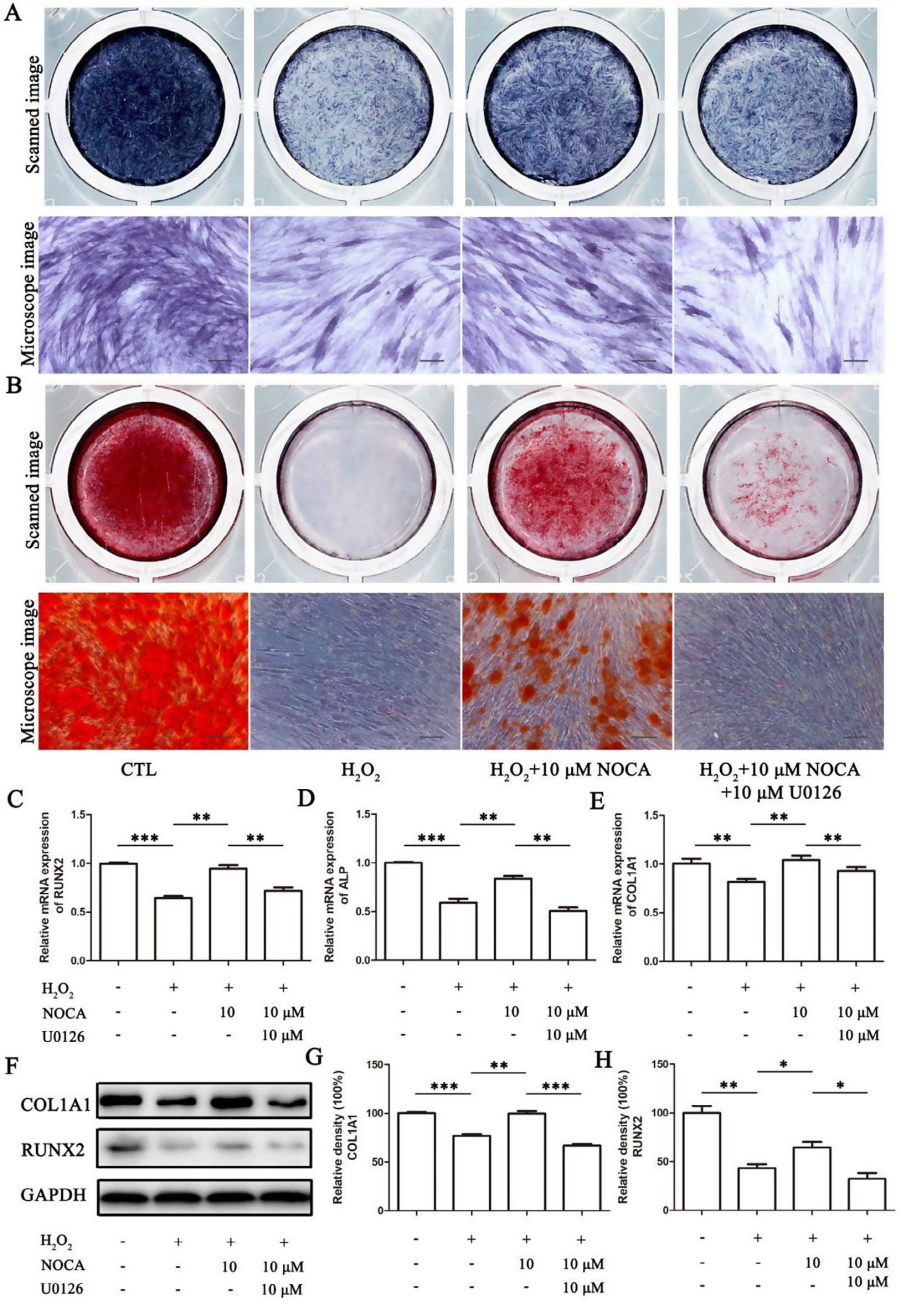
The impact of the ERK pathway on the protection of NOCA in OS-induced impairment of osteogenic differentiation in PDLSCs. Prior to treatment with H_2_O_2_ and NOCA for 24 h using a similar method as described above, PDLSCs was pre-treated with 10 µM U0126 for 1 h. Subsequently, the cells were induced in an osteogenic medium. Osteogenic media served as a control group (CTL). (**A**) ALP activity staining images, and (**B**) ARS staining images were acquired using a scanner and microscope (Scale bar = 100 μm). (**C**-**E**) The mRNA expression levels of RUNX2, ALP, and COL1A1 were analyzed by RT-qPCR. (**F**) The protein expression levels of COL1A1 and RUNX2 were examined by WB analysis. (**G**, **H**) Relative band density of (**F**). *P < 0.05, **P < 0.01, ***P < 0.001 vs. NOCA 0 µM treated group, or NOCA 10 µM treated group, *n* = 3

**Fig. 7:**
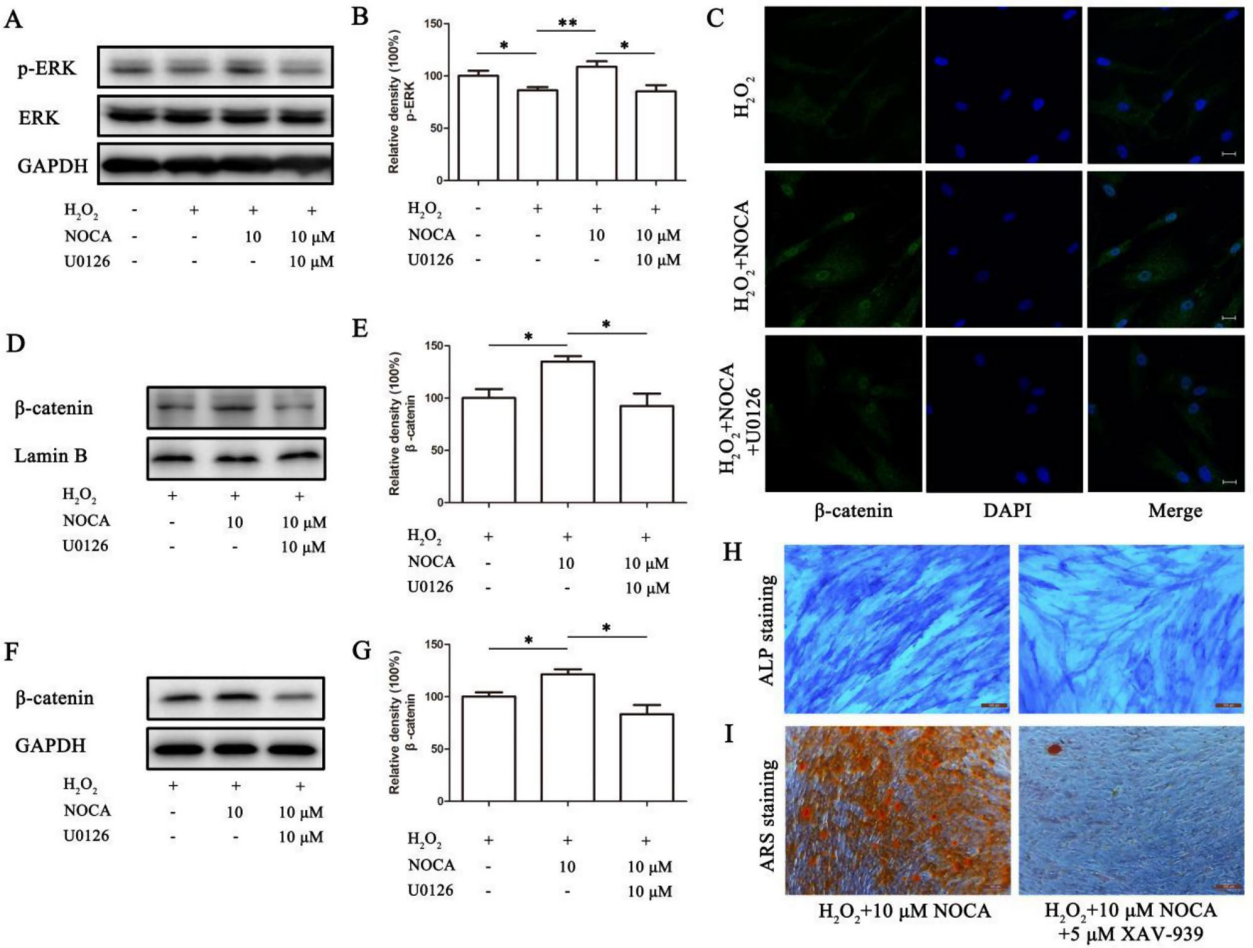
The Wnt/β-catenin pathway serves as the downstream target of ERK pathway. After treatment with U0126, H_2_O_2_ and NOCA using a similar methodology as described above, the cells were cultured in osteogenic medium supplemented with or without 5 µM XAV-939. (**A**) The protein expression levels of p-ERK and ERK. (**B**) Relative band density of (**A**). (**C**) Confocal microscopy was employe to detect the results of immunofluorescence staining of β-catenin (Scale bar = 20 μm). (**D**) The protein levels of β-catenin in the nucleus. (**E**) Relative band density of (**D**). (**F**) The protein levels of β-catenin in the cytoplasm. (**G**) Relative band density of (**F**). (**H**) ALP activity staining images, and (**I**) ARS staining images were acquired using a microscope (Scale bar = 100 μm). *P < 0.05, **P < 0.01 vs. NOCA 0 µM treated group, or NOCA 10 µM treated group, *n* = 3

To further elucidate the role of ERK signaling in NOCA’s protective function, we pre-treated PDLSCs with the ERK inhibitor U0126 for 1 h prior to subjecting them to NOCA and H_2_O_2_ treatment for 24 h. This was followed by osteogenic differentiation induction for 7 and 14 days. Osteogenesis analysis revealed that 10 µM U0126 significantly decreased ALP activity and calcium nodule formation in NOCA-pretreated PDLSCs under H_2_O_2_-induced OS conditions (*p* < 0.05) (Fig. 6A, B). Additionally, RT-qPCR analysis showed downregulation of key osteogenic markers RUNX2, ALP, and COL1A1 upon U0126 treatment (Fig. 6C-H). As ERK is known to influence the Wnt signaling pathway, a downstream target of β-catenin in PDLSC osteogenesis [18], we investigated their potential crosstalk. Immunofluorescence analysis revealed that NOCA treatment significantly augmented the levels of β-catenin in both the nucleus and cytoplasm, as compared to H_2_O_2_ alone (*p* < 0.05). Notably, U0126 treatment significantly reduced the levels of nuclear and cytoplasmic β-catenin in both H_2_O_2_ and NOCA-treated PDLSCs (Fig. 7C-G). Furthermore, blocking the Wnt/β-catenin pathway using 5 µM XAV-939 significantly impaired osteogenesis potential in PDLSCs exposed to H_2_O_2_ and NOCA (Fig. 7H, I), thereby negating the protective effect exerted by NOCA on OS-induced impairment of osteogenic differentiation process. These findings collectively suggest that NOCA protects PDLSCs from OS-induced impairment of osteogenic differentiation through modulation of the ERK/Wnt signaling pathway, with β-catenin nuclear translocation playing a crucial role in this process.

## Discussion

The association between oxidative stress (OS) and periodontal tissue destruction has been reported [2]. Dysregulation of the cellular redox balance, characterized by an imbalance between ROS production and antioxidant defenses, leads to cellular damage and dysfunction. This underscores the therapeutic potential of natural antioxidants, particularly those derived from marine organisms, in combating OS-related periodontal disease [19, 20]. Here, we investigated the antioxidant effect and underlying mechanism of NOCA, a compound isolated from the marine sponge *Dysidea* sp., on H_2_O_2_-induced OS in human periodontal ligament stem cells (PDLSCs).

PDLSCs represent a distinct subset of mesenchymal stem cells residing within periodontal tissues, distinguished by their remarkable capacity for self-renewal and potential to regenerate periodontal tissues [21]. Since the pioneering work of Seo et al. [21]. PDLSCs have gained widespread utilization in investigating periodontal diseases due to their advantages in terms of safety and ethical concerns. Following the protocol outlined by published articles [10–12], we successfully isolated PDLSCs from healthy human individuals and confirmed their typical mesenchymal stem cell characteristics through phenotypic characterization and assessment of differentiation potential. Although PDLSCs have been extensively investigated for nearly two decades, their clinical application remains elusive due to their inherent susceptibility to dysfunction upon exposure to external stimuli.

OS, characterized by an imbalance between ROS production and antioxidant defense, significantly contributes to periodontal tissue destruction [3]. Que is widely recognized as a potent antioxidant and has been extensively investigated for its ability to enhance the functionality of PDLSCs and other cell lines impaired by OS through effective clearance of excessive ROS and augmentation of antioxidant factors [13, 14, 22]. Consequently, Que was employed in this study as a reference control to investigate small molecules with therapeutic potential. We then investigated the antioxidant effects of NOCA in PDLSCs exposed to H_2_O_2_-induced OS. The evaluation revealed minimal cytotoxicity of NOCA in PDLSCs and other cell lines. Moreover, NOCA potently reduces ROS levels compared to H_2_O_2_-treated controls. This ROS scavenging ability is further bolstered by upregulation of key antioxidant enzymes, superoxide dismutase (SOD) and catalase (CAT) [23, 24]. NOCA treatment increases SOD activity and CAT activity, effectively neutralizing superoxide anions and hydrogen peroxide, respectively. SIRT1, a nicotinamide adenine dinucleotide-dependent deacetylase, is involved in OS response by reducing ROS levels and regulating several antioxidant genes, such as SOD and CAT [25, 26]. Previous studies reported that SIRT1 activation for repairing OS-induced cellular dysfunction involves multiple defense mechanisms [25, 26]. Our studies demonstrated that NOCA reversed the impaired expression of SIRT1 in PDLSCs caused by H_2_O_2_ in PDLSCs. In our previous work, we screened around 36 compounds isolated from Dysidea sp. Marine Sponge, many of which possess phenolic hydroxyl groups commonly associated with antioxidant activity. Notably, NOCA was the only compound that demonstrated a protective effect on PDLSCs against H_2_O_2_-induced OS (Fig. S2). This selectivity suggests that NOCA’s interaction with H_2_O_2_ may involve direct chemical neutralization, in addition to any intracellular reactions it may trigger. We hypothesize that NOCA’s primary protective mechanism involves modulating intracellular pathways. The pretreatment period allowed NOCA to potentially interact with cellular components, preparing the cells to counteract the subsequent oxidative challenge.

While most studies exploring OS damage to PDLSCs focus on cell viability and osteogenic potential, both vital factors in periodontal tissue regeneration, the underlying mechanisms remain incompletely understood [3]. Counteracting excessive OS is crucial for restoring H_2_O_2_-induced damage to cell viability and osteogenic differentiation in PDLSCs [3]. Prior studies have demonstrated that manipulating key regulators like Tripartite Motif 16 or recombinant Klotho protein can alleviate OS, thereby rescuing PDLSC function [27, 28]. Our study builds upon these findings by revealing that NOCA not only enhances cell viability but also promotes osteogenic differentiation of PDLSCs specifically under H_2_O_2_-induced OS. Notably, NOCA exhibits no effect on osteogenic potential under normal conditions (data not shown), suggesting its targeted action against OS-mediated dysfunction.

**Fig. 8:**
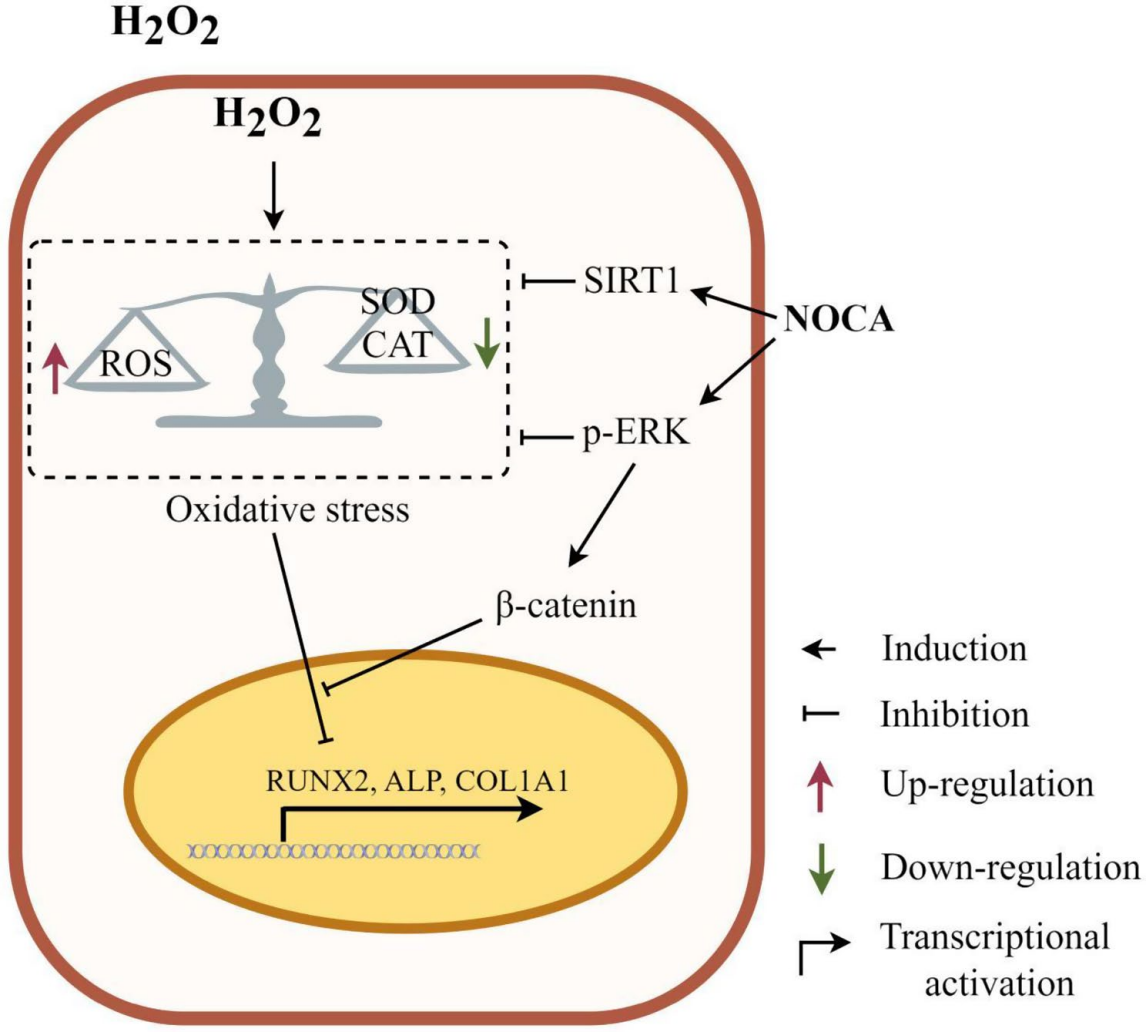
NOCA alleviates H_2_O_2_-induced oxidative stress and restores the damaged osteogenic potential of PDLSCs through preserved ERK/Wnt signaling pathway

While the Akt signaling pathway plays a crucial role in antioxidant defense by stimulating the nuclear factor erythroid 2-related factor2 (Nrf2) pathway [28], our data suggest NOCA’s protective effect in PDLSCs is mediated primarily through the ERK signaling cascade. Pretreatment with NOCA showed no significant impact on Akt signaling, but interestingly, it significantly upregulated p-ERK, the active form of the ERK pathway. This finding aligns with previous reports demonstrating that activation of p-ERK alleviates H_2_O_2_-induced damage in PDLSCs [29]. Moreover, our study further corroborates this link by showing that H_2_O_2_ suppressed p-ERK levels, while NOCA pretreatment reversed this suppression, restoring p-ERK activity. Importantly, blocking the ERK pathway using U0126 abolished NOCA’s protective effect on osteogenic differentiation, underscoring the critical role of ERK signaling in NOCA’s mechanism of action. Therefore, our findings suggest that NOCA primarily modulates the ERK signaling pathway, rather than Akt, to counteract OS-induced impairment of osteogenic differentiation in PDLSCs. This specific targeting of the ERK pathway highlights a potentially unique mechanism of action for NOCA, warranting further investigation of its downstream targets and potential therapeutic applications.

The Wnt/β-catenin signaling pathway plays a vital role in protecting against oxidative damage. H_2_O_2_ disrupts this pathway by suppressing β-catenin, leading to impaired survival and bone formation capabilities [30]. Conversely, activating the Wnt/β-catenin pathway safeguards PDLSCs from these detrimental effects [31]. Notably, this pathway is downstream of ERK signaling and crucial for PDLSC proliferation, differentiation, and bone tissue homeostasis [18]. Our study reveals NOCA’s potent protective effect against H_2_O_2_-induced damage in PDLSCs through its modulation of the ERK/Wnt axis: (a) NOCA enhances β-catenin expression and promotes its nuclear translocation under OS. (b) Blocking the Wnt pathway significantly diminishes NOCA’s protective effect on PDLSC osteogenic differentiation under H_2_O_2_-induced OS. (c) ERK pathway inhibition also prevents NOCA from increasing nuclear and cytoplasmic levels of β-catenin. These findings demonstrate that NOCA’s ability to improve osteogenic potential in PDLSCs under OS hinges primarily on its preservation of the ERK/Wnt signaling pathway. This targeted action presents a unique mechanism of action and highlights NOCA’s potential as a promising therapeutic candidate for alleviating OS-related periodontal diseases.

Despite these significant in vitro findings, the translational potential of NOCA as a therapeutic agent for OS-related periodontal diseases cannot be fully ascertained without in vivo studies. In animal models, factors such as bioavailability, pharmacokinetics, and the interaction of NOCA with the host’s immune system and other physiological processes can be assessed. These are critical considerations for the development of NOCA as a clinical therapeutic.

In conclusion, our study demonstrates that NOCA effectively counteracts H_2_O_2_-induced oxidative stress in PDLSCs, significantly restoring their impaired osteogenic potential. This protective effect appears to be mediated, at least in part, by NOCA’s ability to preserve the ERK/Wnt signaling pathway, a key regulator of cell survival and differentiation. These findings suggest NOCA’s potential as a promising natural therapeutic agent for alleviating periodontal tissue destruction associated with OS. However, further in vivo studies are necessary to validate NOCA’s efficacy and safety in a physiological context and fully elucidate its therapeutic potential for clinical applications. Our future research will focus on these critical next steps.

### Electronic supplementary material

Below is the link to the electronic supplementary material.

Supplementary Material 1

## Data Availability

All data generated or analyzed during this study are included in this published article.
