# Supplementary material for: Nocardamine mitigates cellular dysfunction induced by oxidative stress in periodontal ligament stem cells
Source: Stem Cell Res Ther. 2024 Aug 7;15:247. doi: 10.1186/s13287-024-03812-2 (PMC11305061; doi:10.1186/s13287-024-03812-2)
Supplement: Supplementary file 1 — Supplementary Material 1 [file 13287_2024_3812_MOESM1_ESM.pdf]

The screenshot's location is indicated by the red box.

(A) Associated with Figure 3I

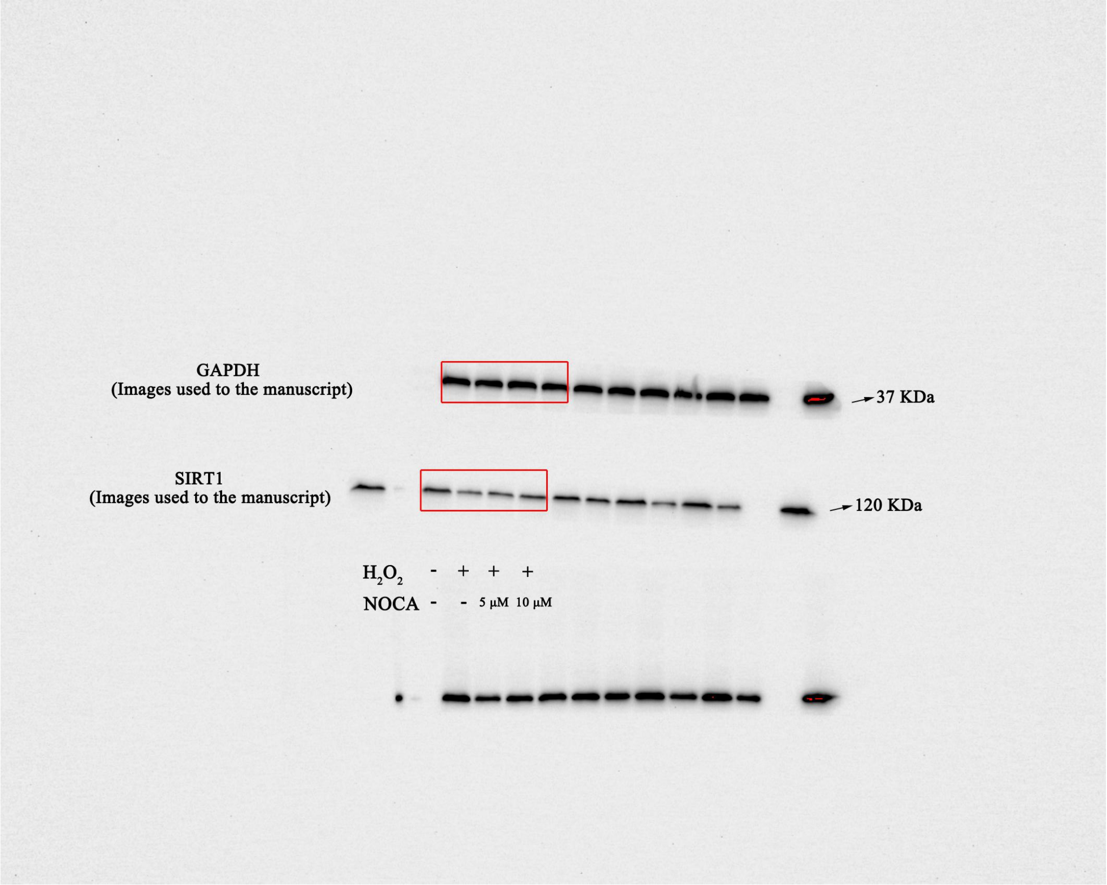

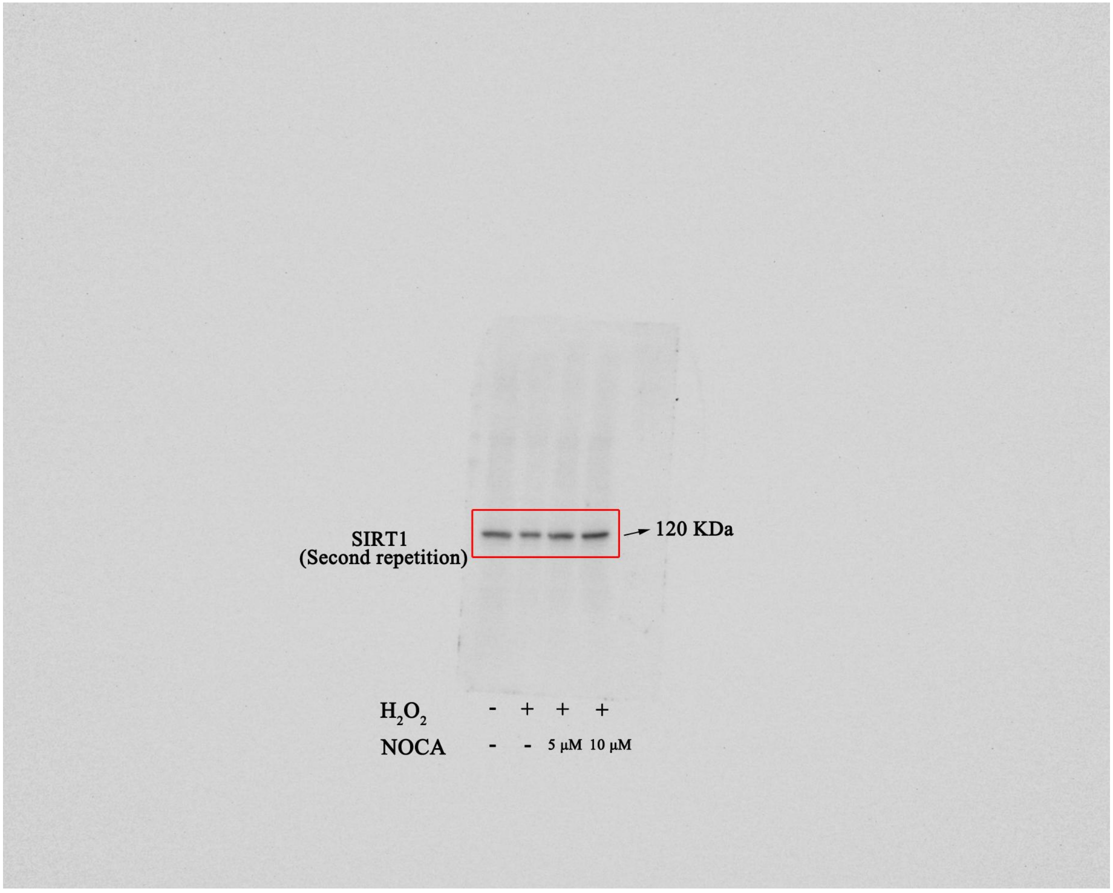
SIRT1
(Second repetition)
120 KDa
H_2O_2 - + + +
NOCA - - 5 μM 10 μM

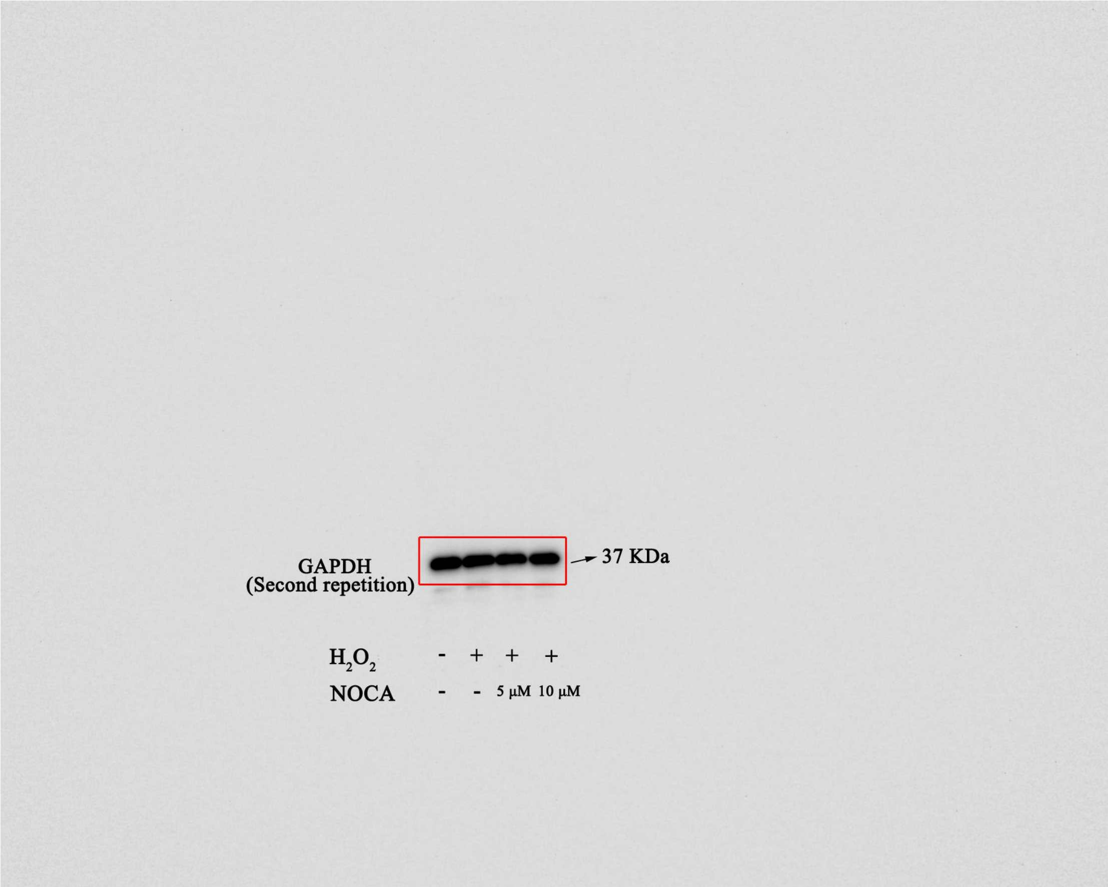
GAPDH
(Second repetition)
37 KDa
H_2O_2 - + + +
NOCA - - 5 μM 10 μM

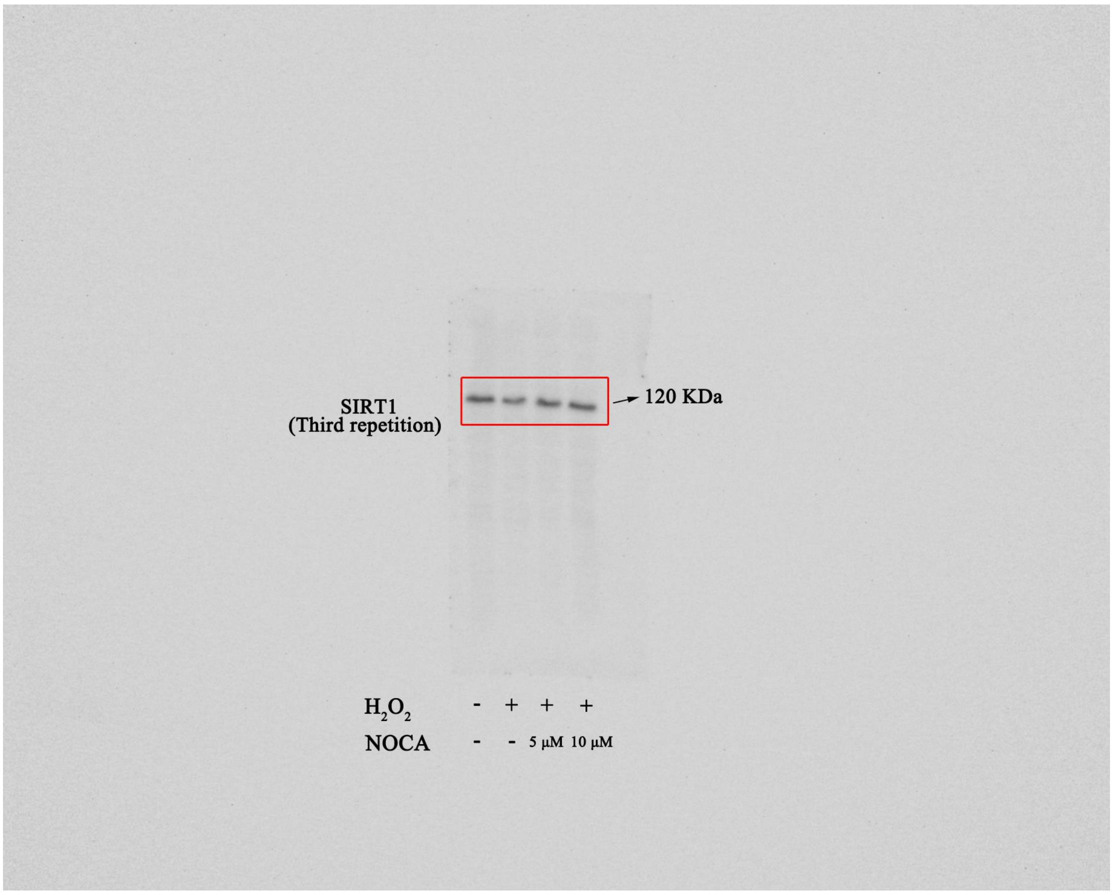
SIRT1
(Third repetition)
120 KDa
H2O2 - + + +
NOCA - - 5 μM 10 μM

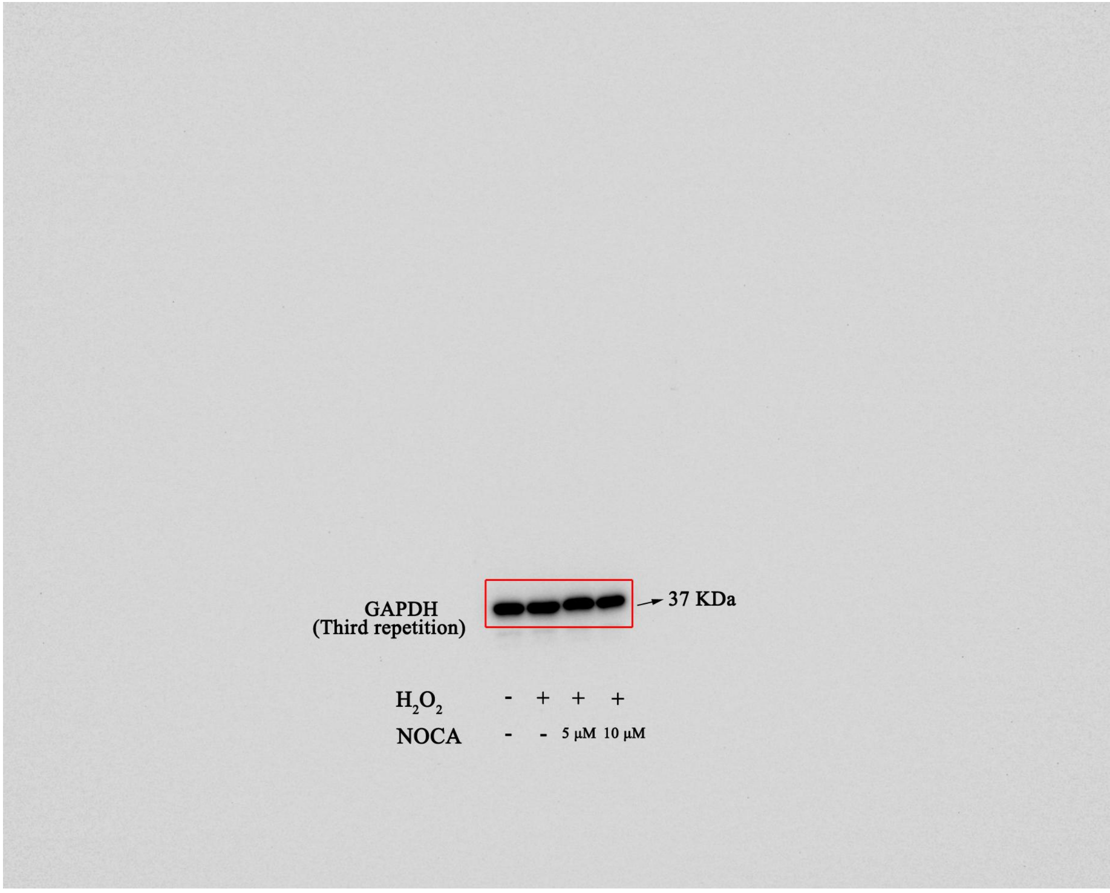
GAPDH
(Third repetition)
37 KDa
H2O2 - + + +
NOCA - - 5 μM 10 μM

(B) Associated with Figure 4F

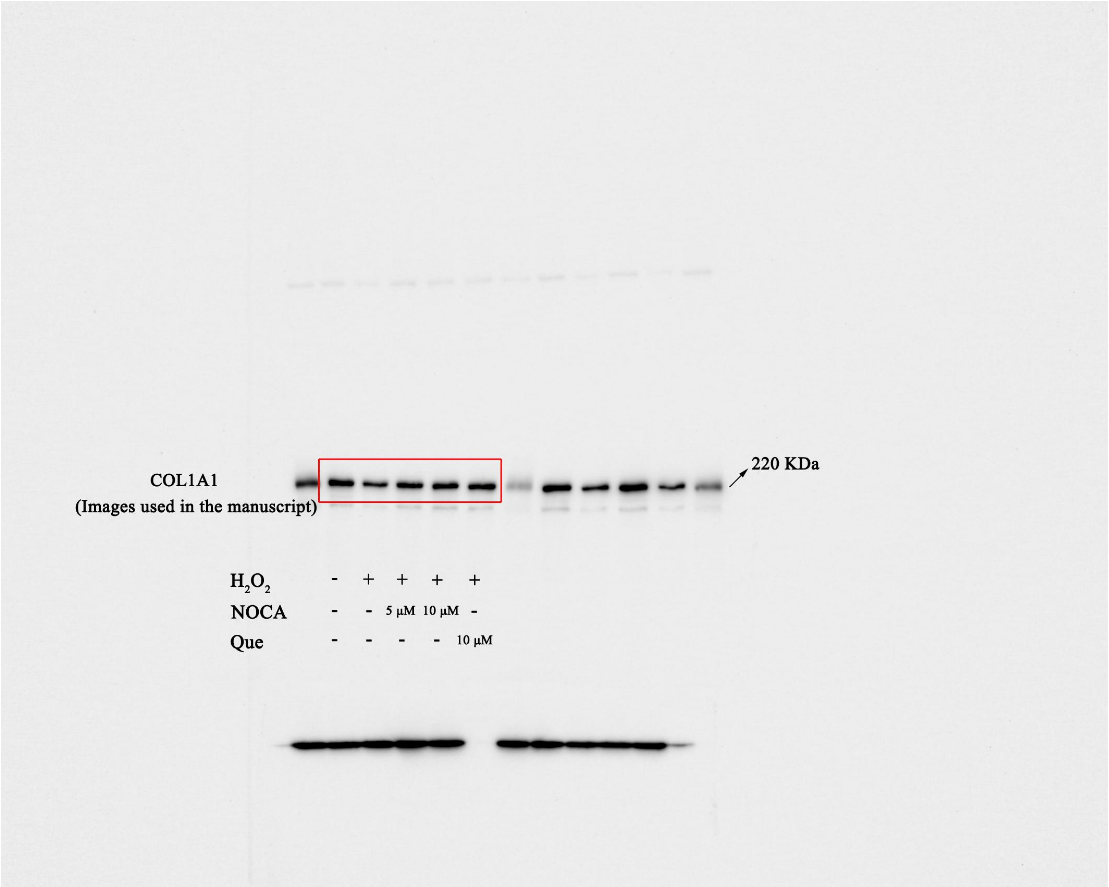

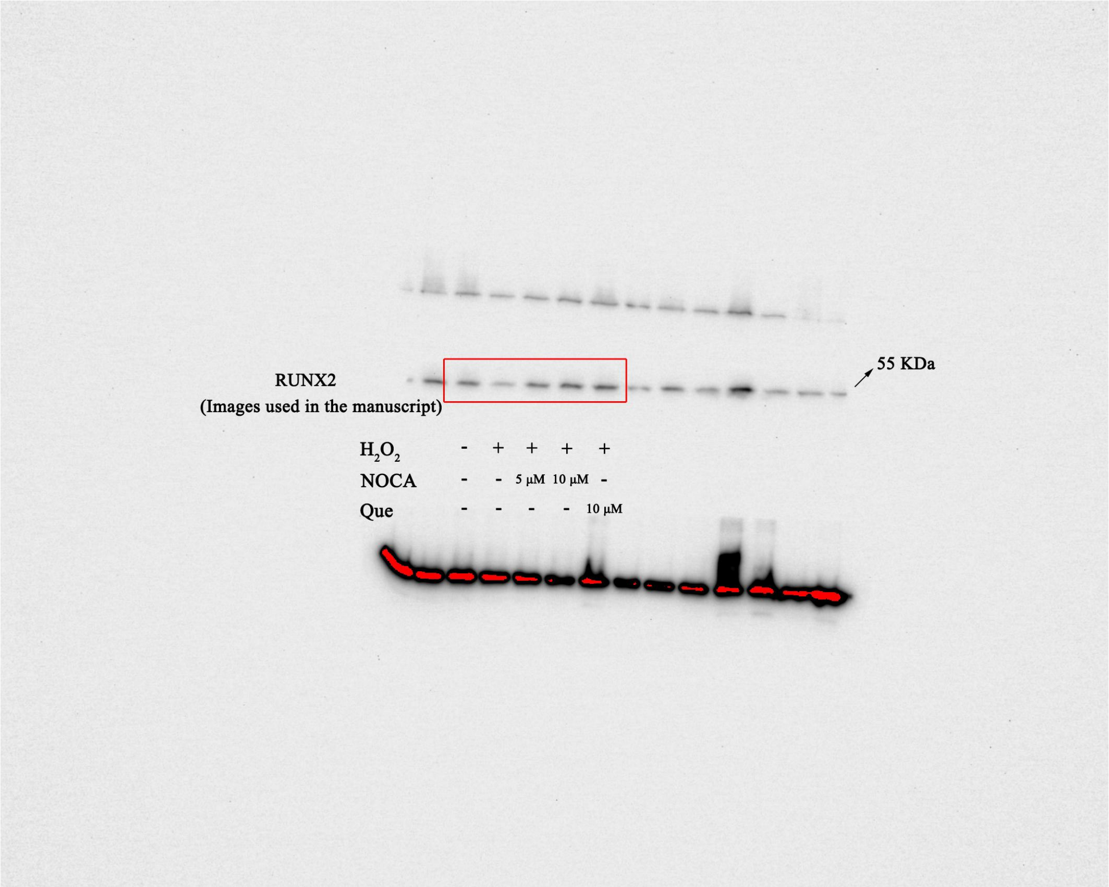
RUNX2
(Images used in the manuscript)
55 KDa
H2O2 - + + + +
NOCA - - 5 μM 10 μM -
Que - - - - 10 μM

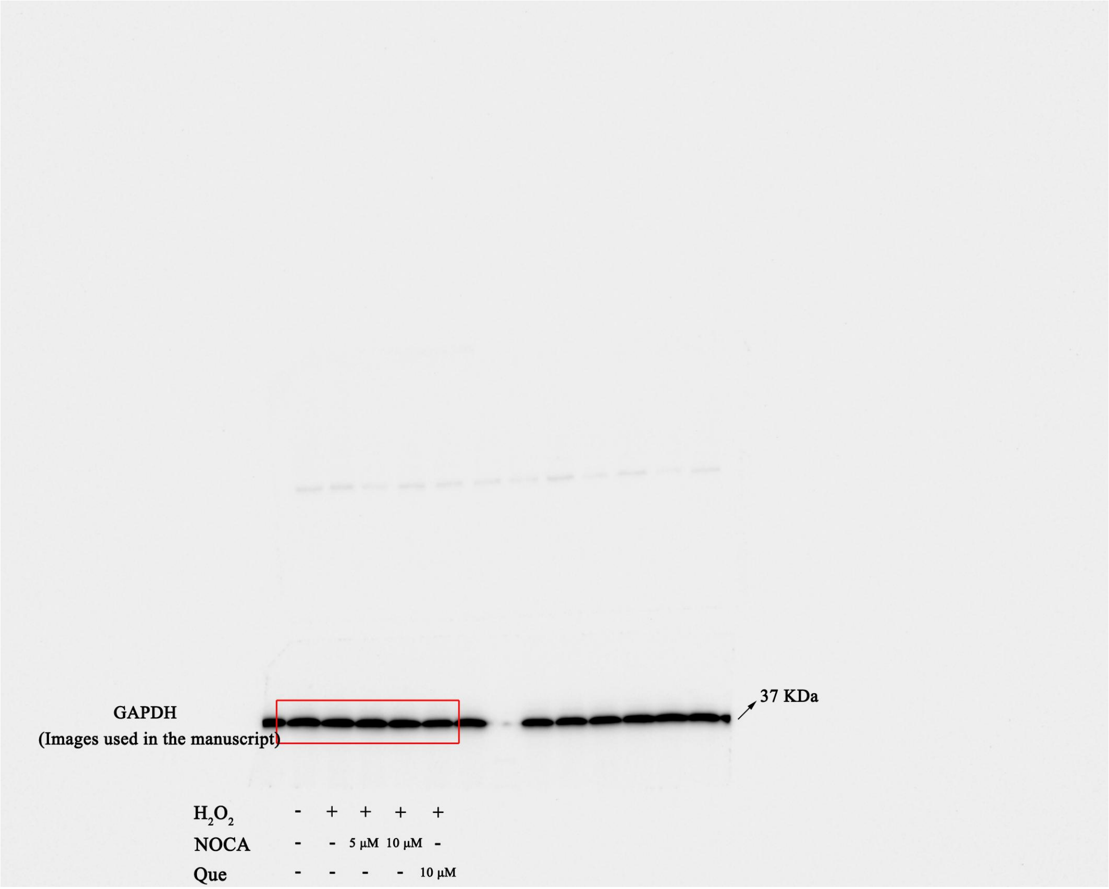
GAPDH
(Images used in the manuscript)
37 KDa
H2O2 - + + + +
NOCA - - 5 μM 10 μM -
Que - - - - 10 μM

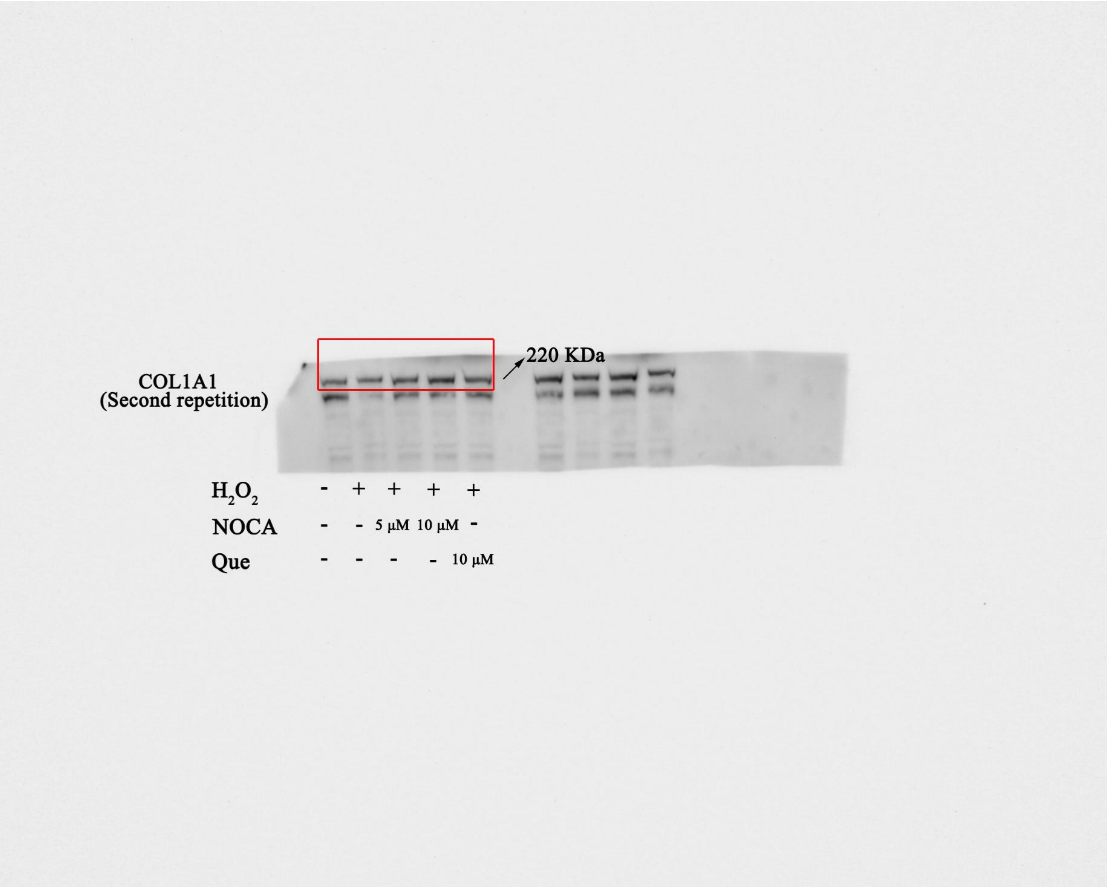
COL1A1
(Second repetition)
220 KDa
H_2O_2 - + + + +
NOCA - - 5 μM 10 μM -
Que - - - - 10 μM

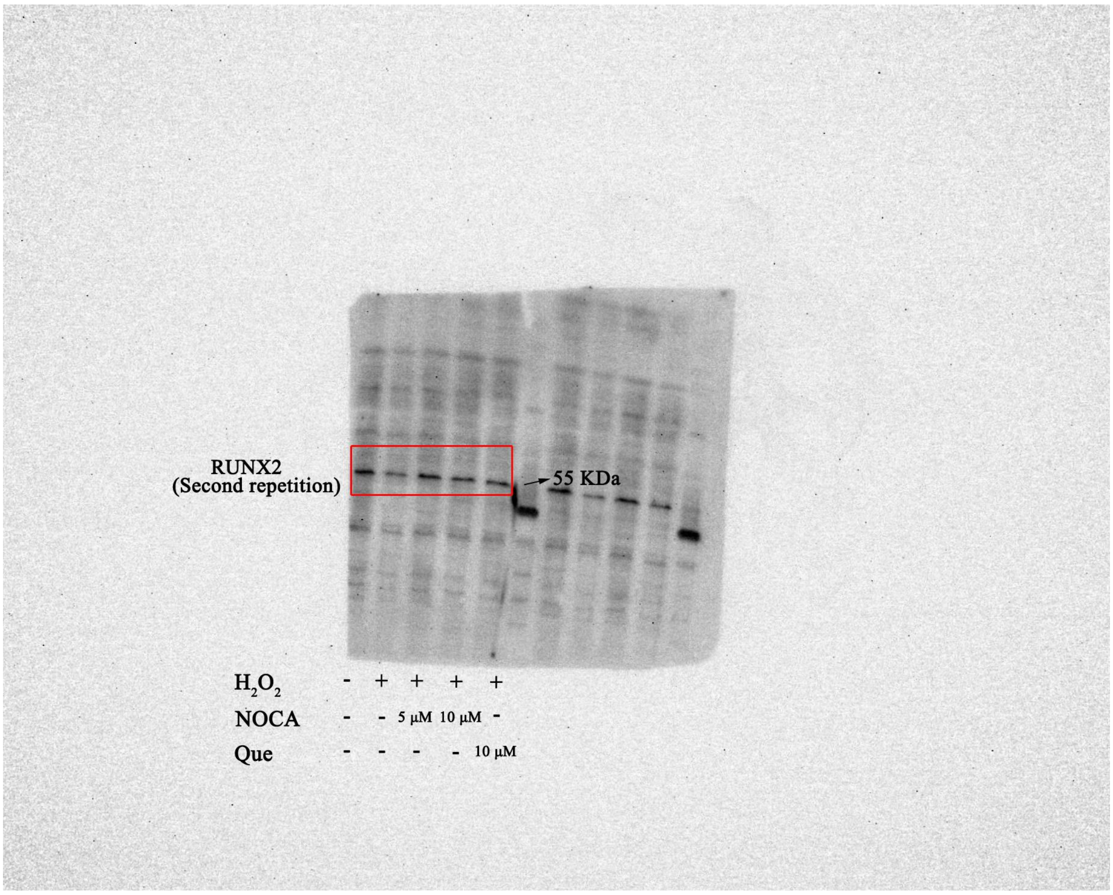
RUNX2
(Second repetition)
55 KDa
H_2O_2 - + + + +
NOCA - - 5 μM 10 μM -
Que - - - - 10 μM

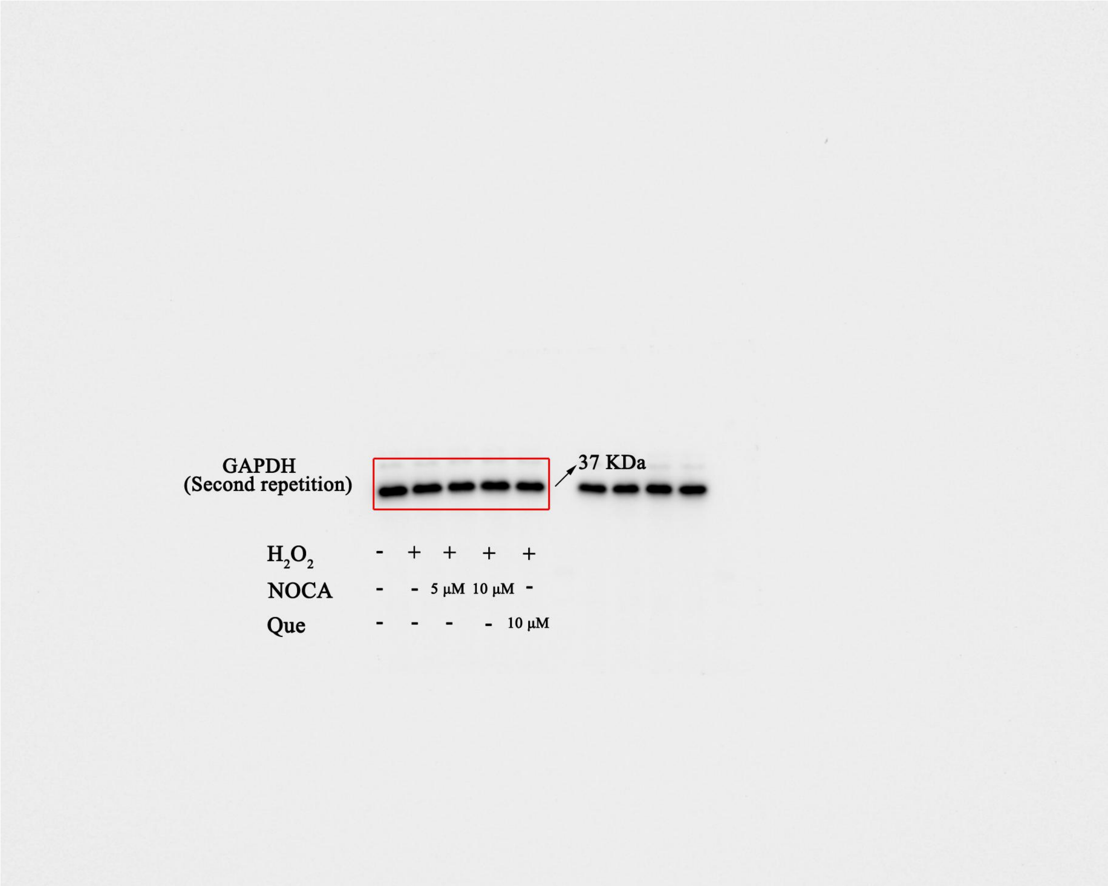
GAPDH
(Second repetition)
37 KDa
H_2O_2 - + + + +
NOCA - - 5 μM 10 μM -
Que - - - - 10 μM

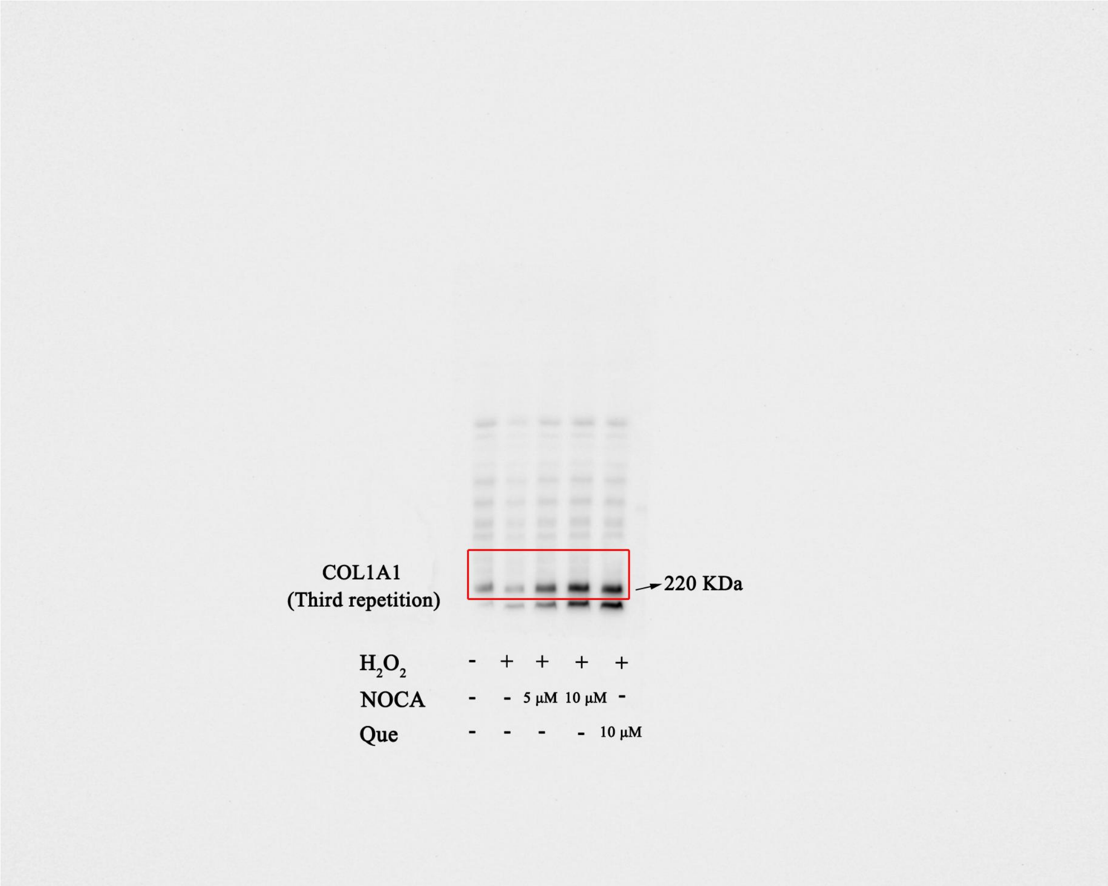
COL1A1
(Third repetition)
220 KDa
H_2O_2 - + + + +
NOCA - - 5 μM 10 μM -
Que - - - - 10 μM

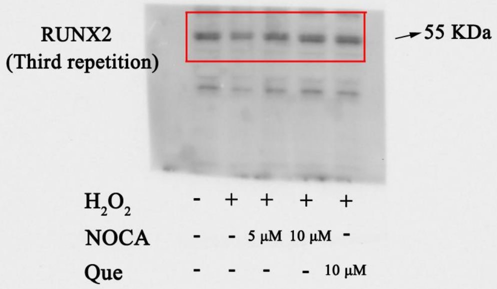
RUNX2
(Third repetition)
55 KDa
H2O2 - + + + +
NOCA - - 5 μM 10 μM -
Que - - - - 10 μM

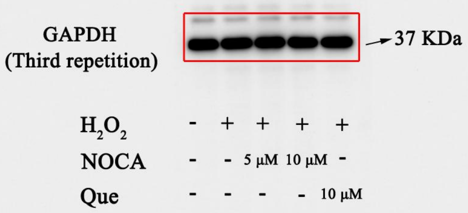
GAPDH
(Third repetition)
37 KDa
H2O2 - + + + +
NOCA - - 5 μM 10 μM -
Que - - - - 10 μM

(C) Associated with Figure 5A, C

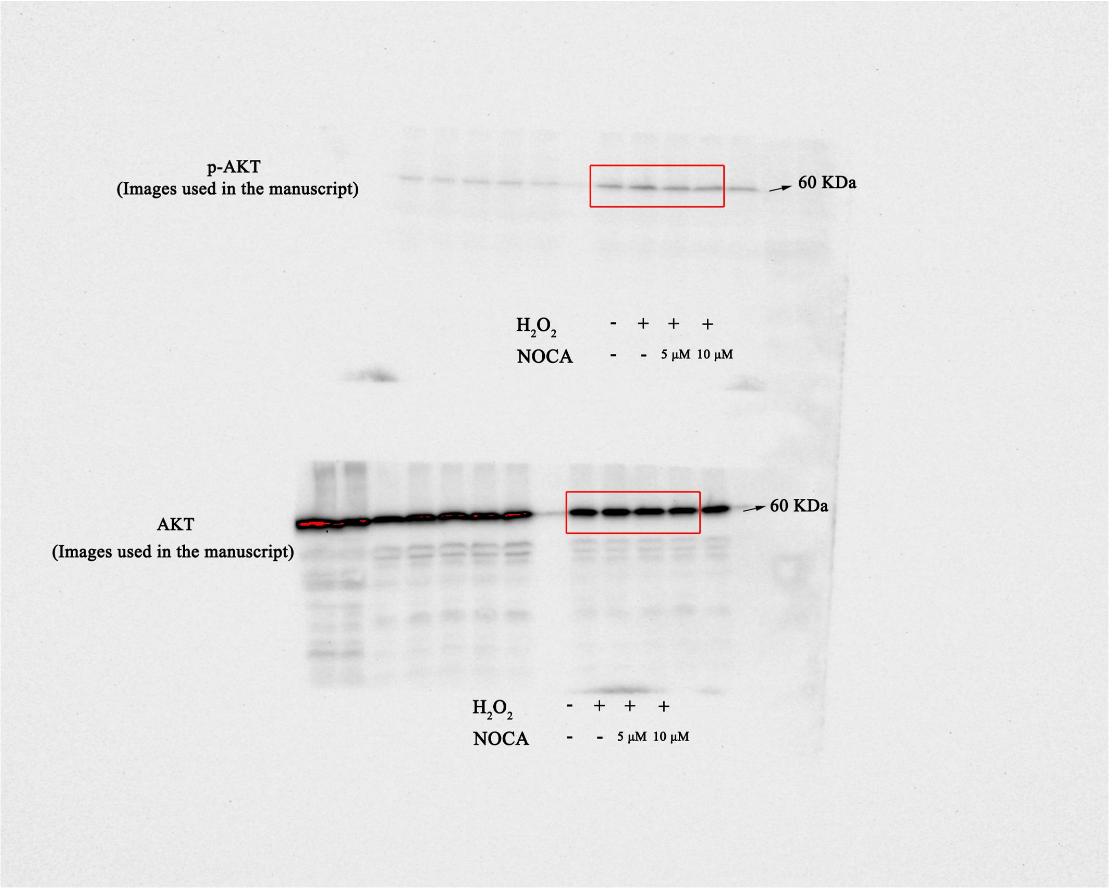

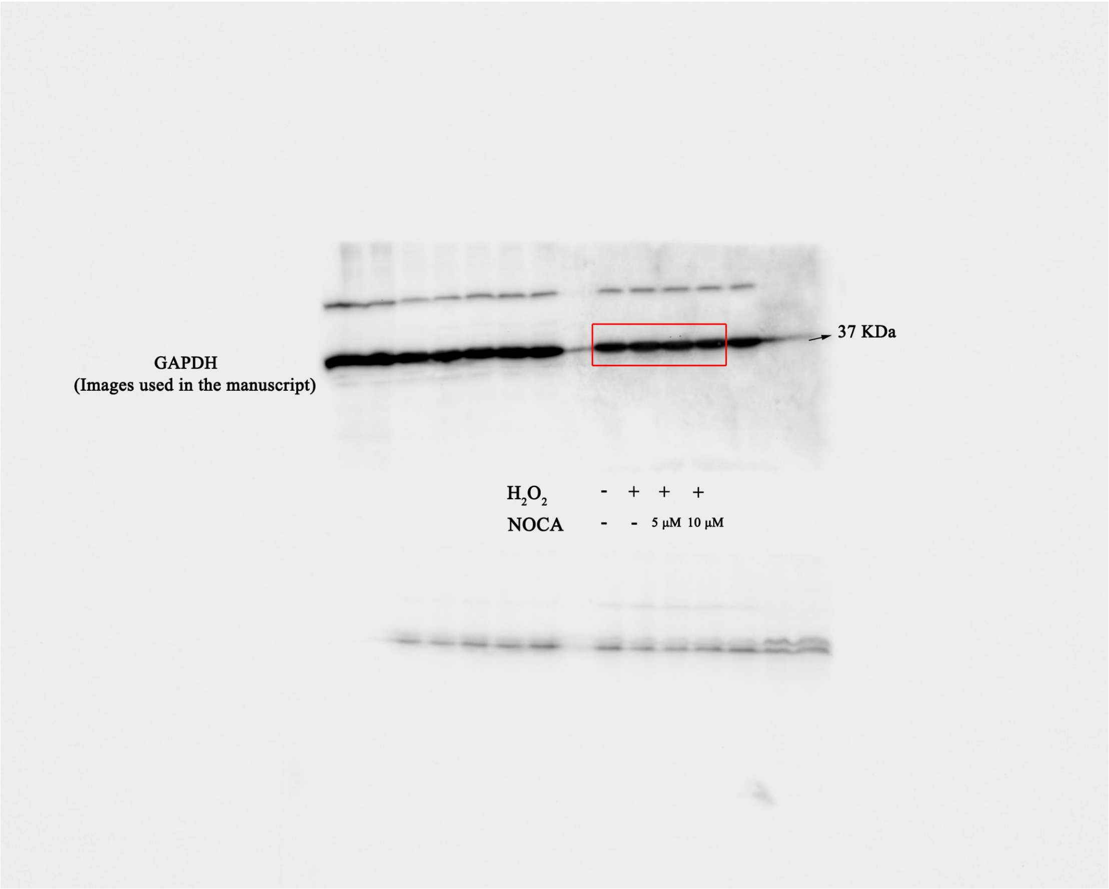
GAPDH
(Images used in the manuscript)
37 KDa
H2O2 - + + +
NOCA - - 5 μM 10 μM

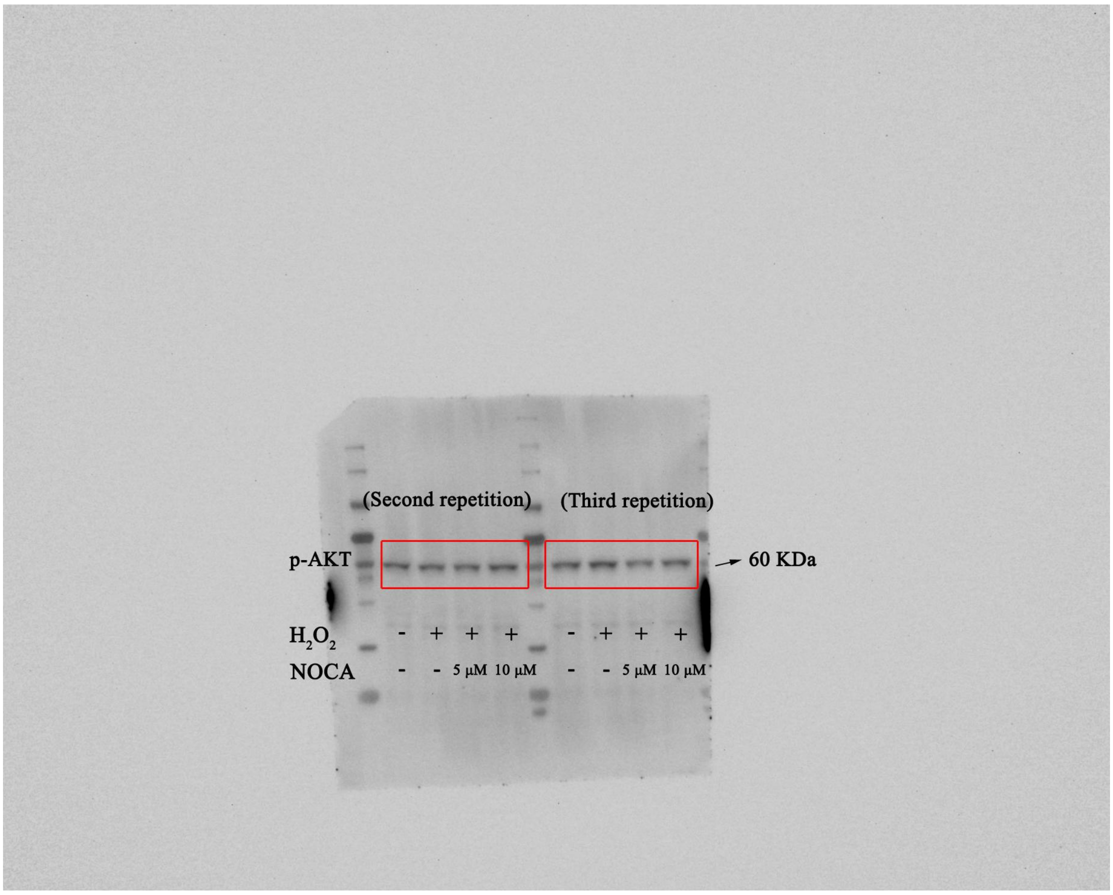
(Second repetition)
(Third repetition)
p-AKT
60 KDa
H2O2 - + + + - + + +
NOCA - - 5 μM 10 μM - - 5 μM 10 μM

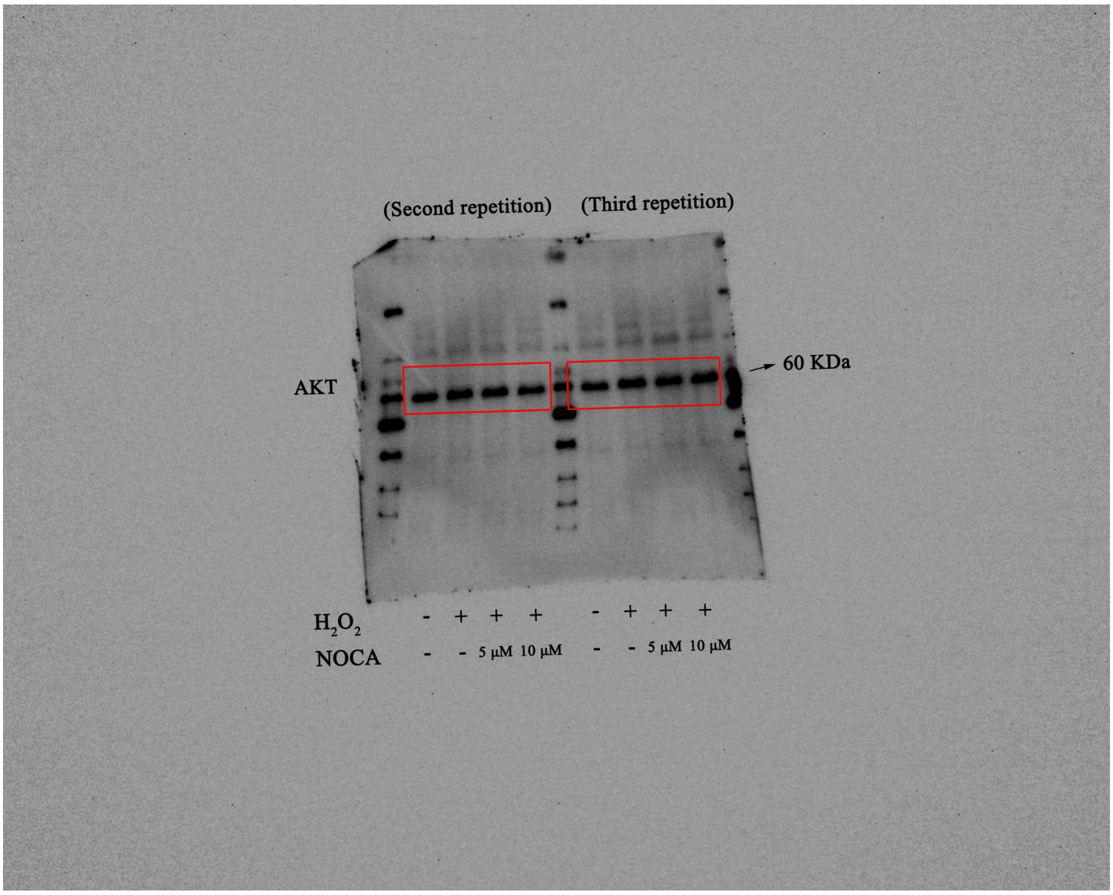

(Second repetition)
(Third repetition)
AKT
60 KDa
H_2O_2 - + + + - + + +
NOCA - - 5 μM 10 μM - - 5 μM 10 μM

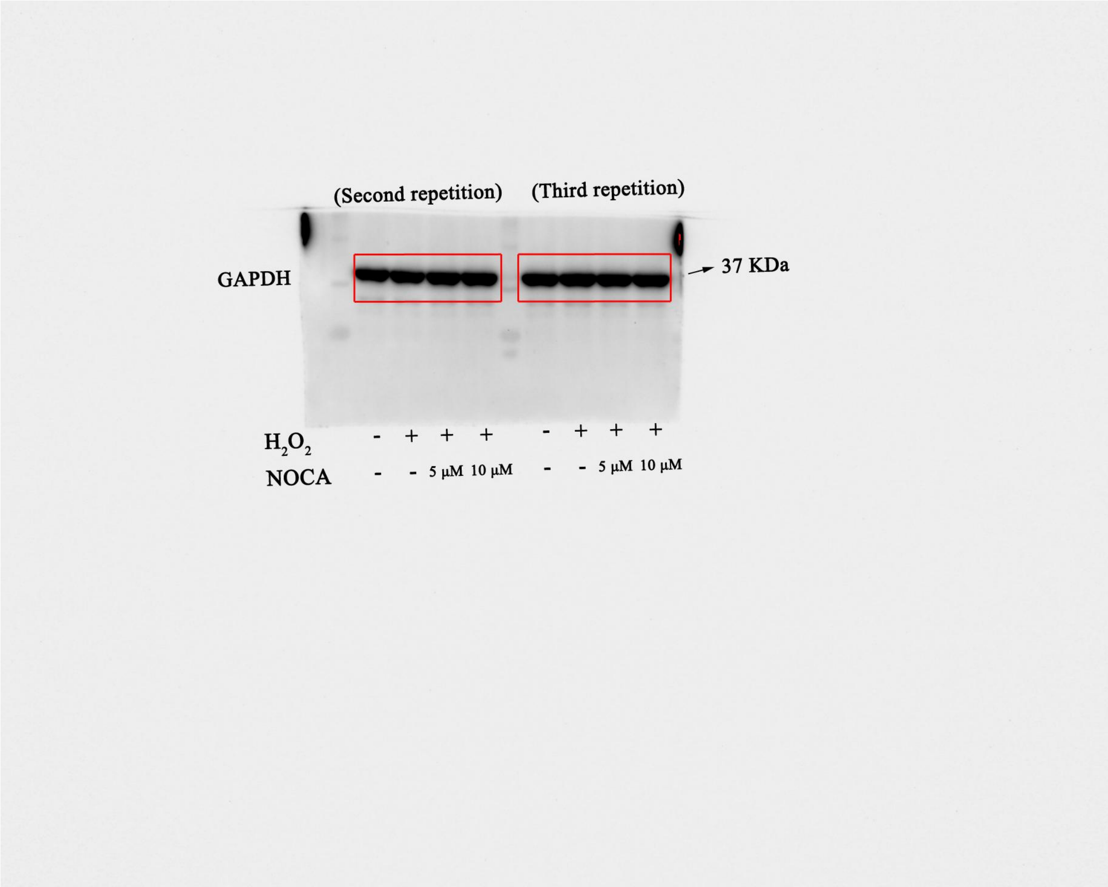

(Second repetition)
(Third repetition)
GAPDH
37 KDa
H_2O_2 - + + + - + + +
NOCA - - 5 μM 10 μM - - 5 μM 10 μM

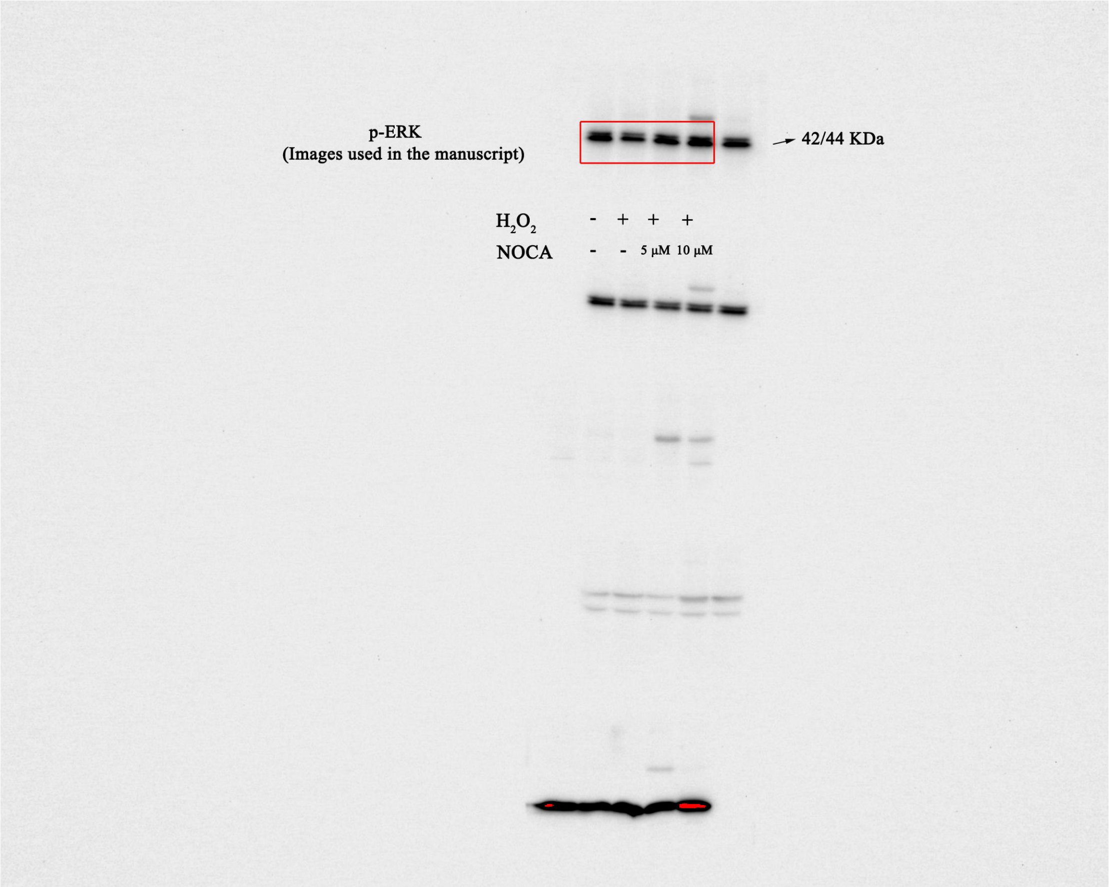
p-ERK
(Images used in the manuscript)
42/44 KDa
H_2O_2 - + + +
NOCA - - 5 μM 10 μM

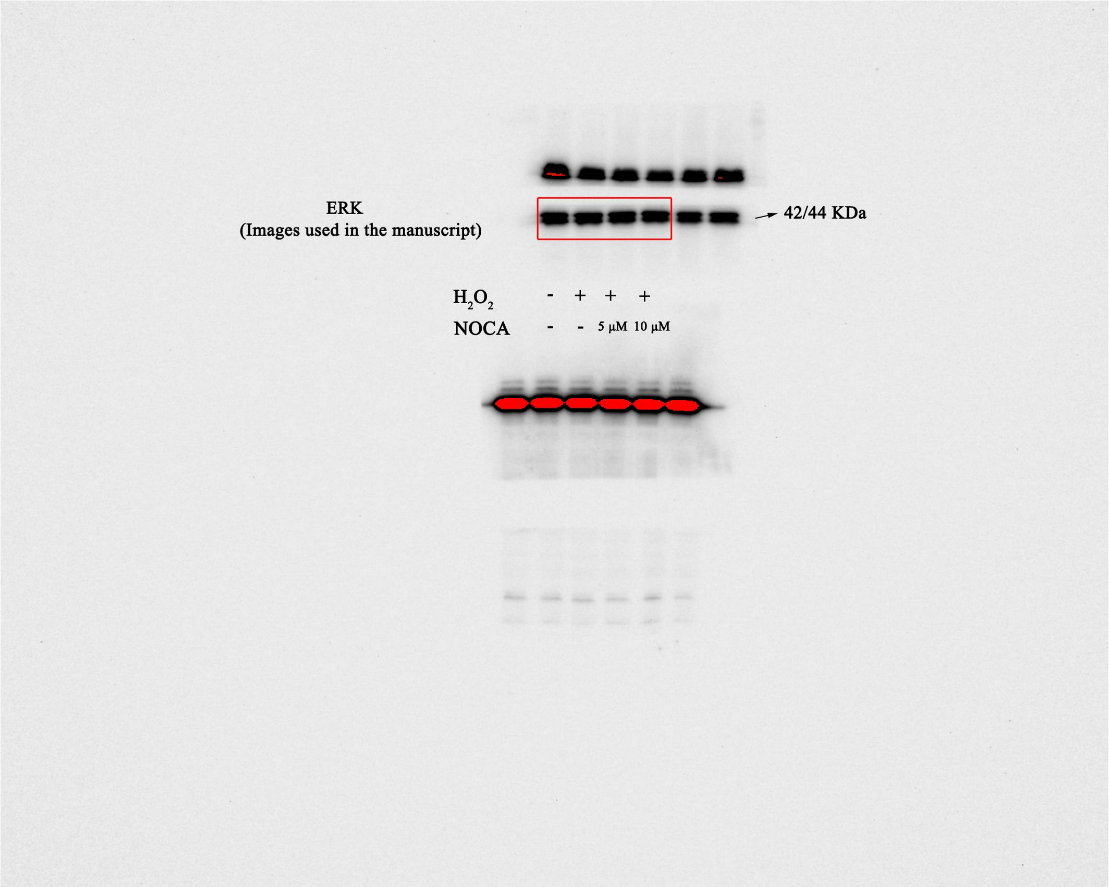
ERK
(Images used in the manuscript)
42/44 KDa
H_2O_2 - + + +
NOCA - - 5 μM 10 μM

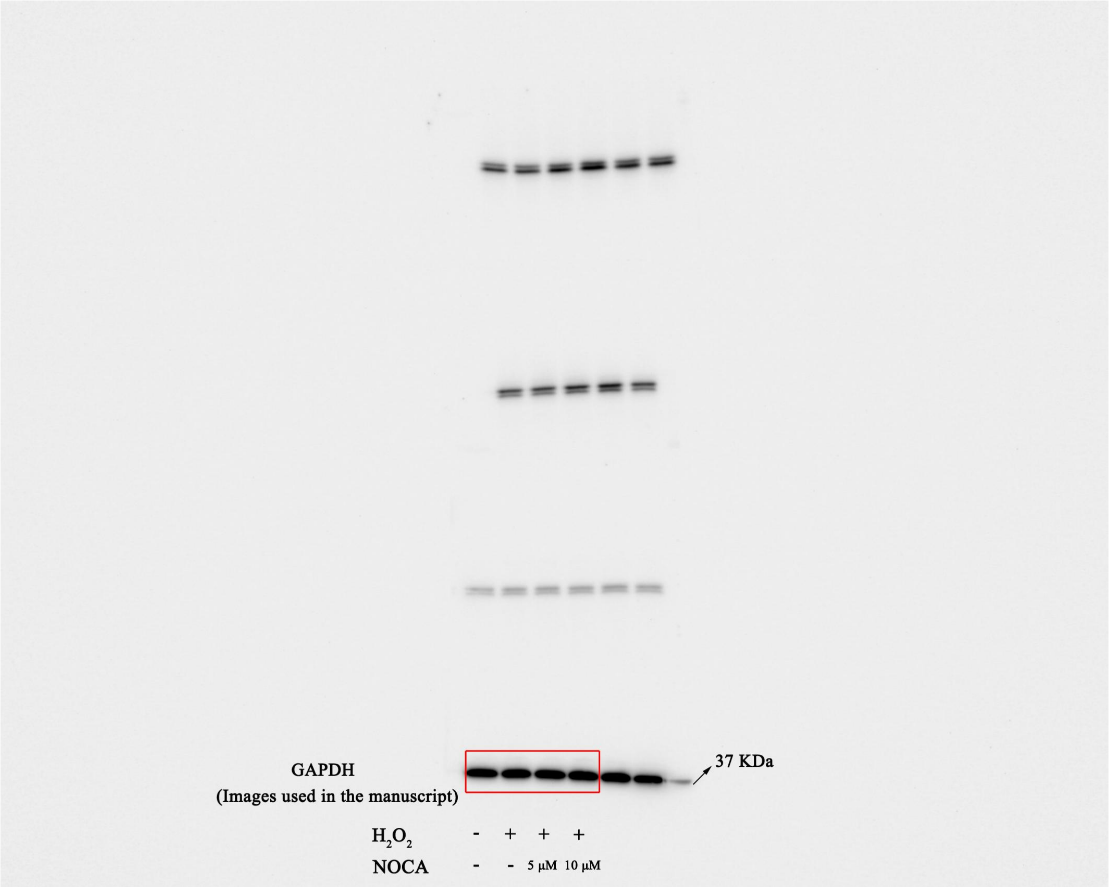

GAPDH
(Images used in the manuscript)
37 KDa
H_2O_2 - + + +
NOCA - - 5 μM 10 μM

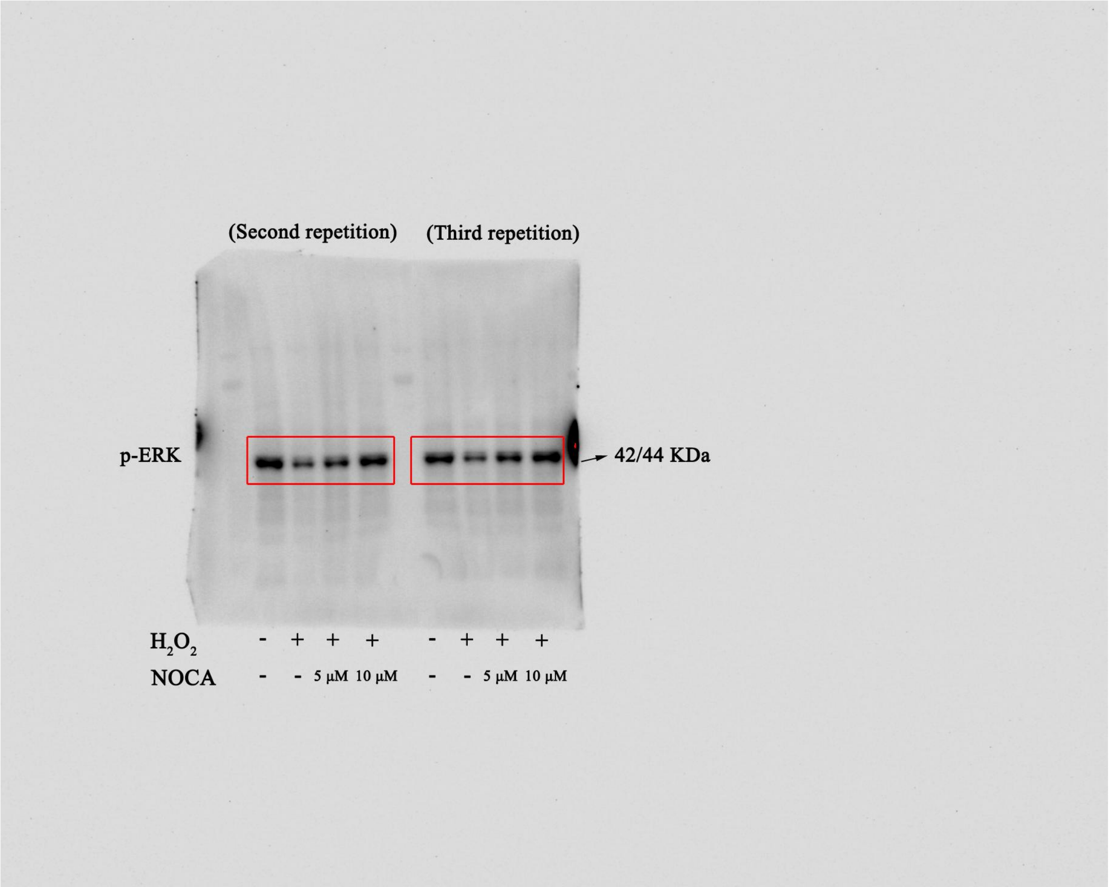

(Second repetition)
(Third repetition)
p-ERK
42/44 KDa
H_2O_2 - + + + - + + +
NOCA - - 5 μM 10 μM - - 5 μM 10 μM

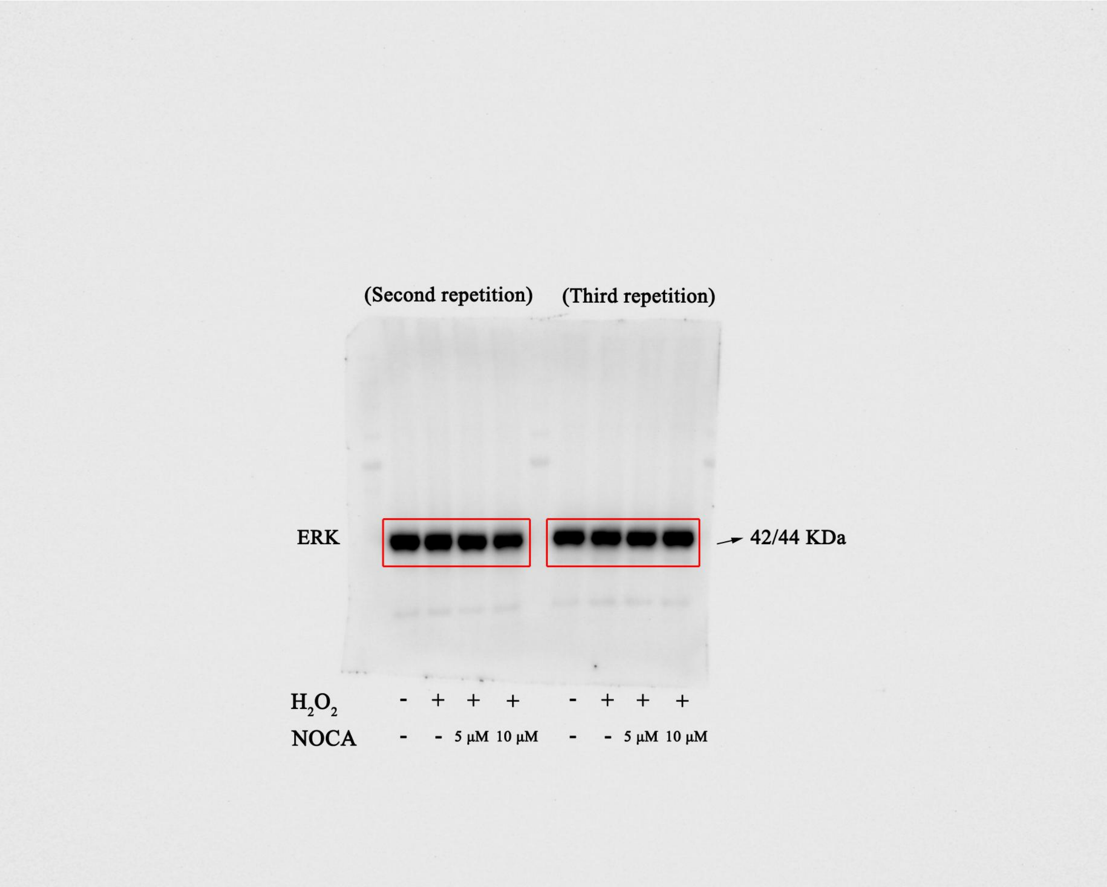
(Second repetition)
(Third repetition)
ERK
42/44 KDa
H_2O_2 - + + + - + + +
NOCA - - 5 μM 10 μM - - 5 μM 10 μM

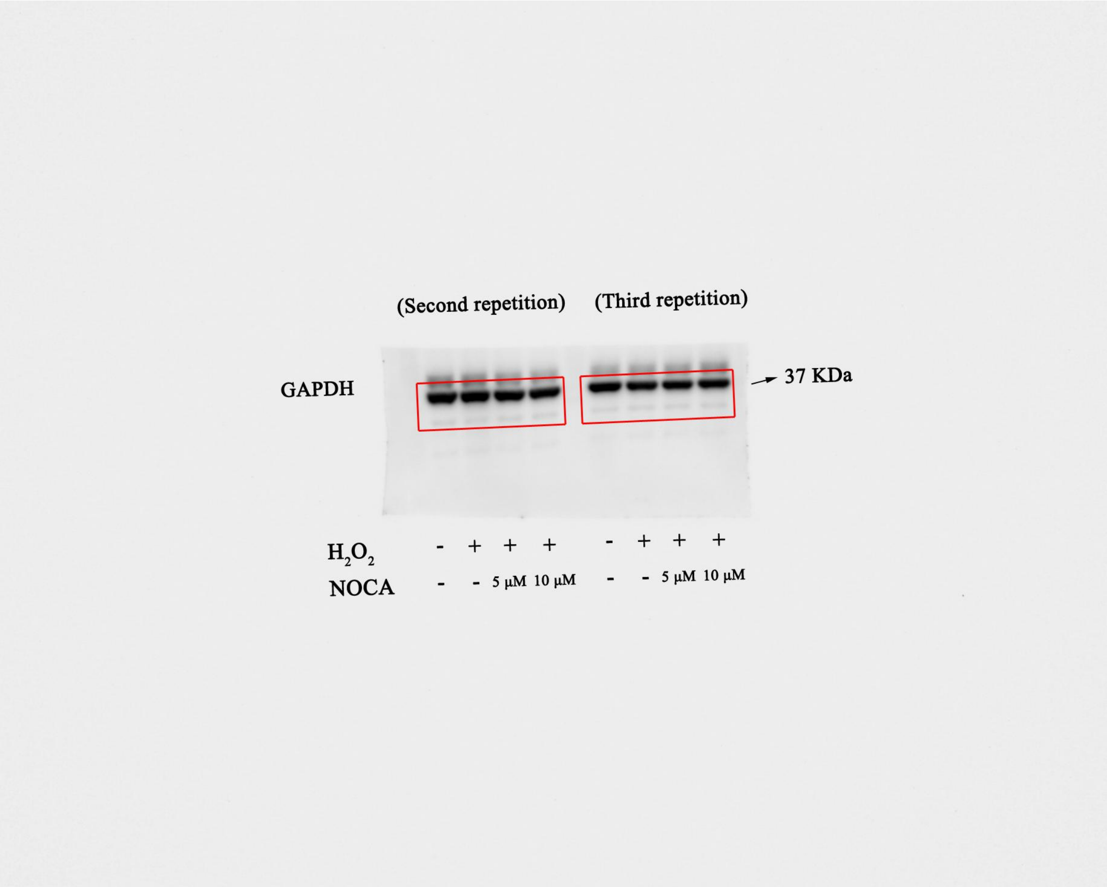
(Second repetition)
(Third repetition)
GAPDH
37 KDa
H_2O_2 - + + + - + + +
NOCA - - 5 μM 10 μM - - 5 μM 10 μM

(D) Associated with Figure 6F

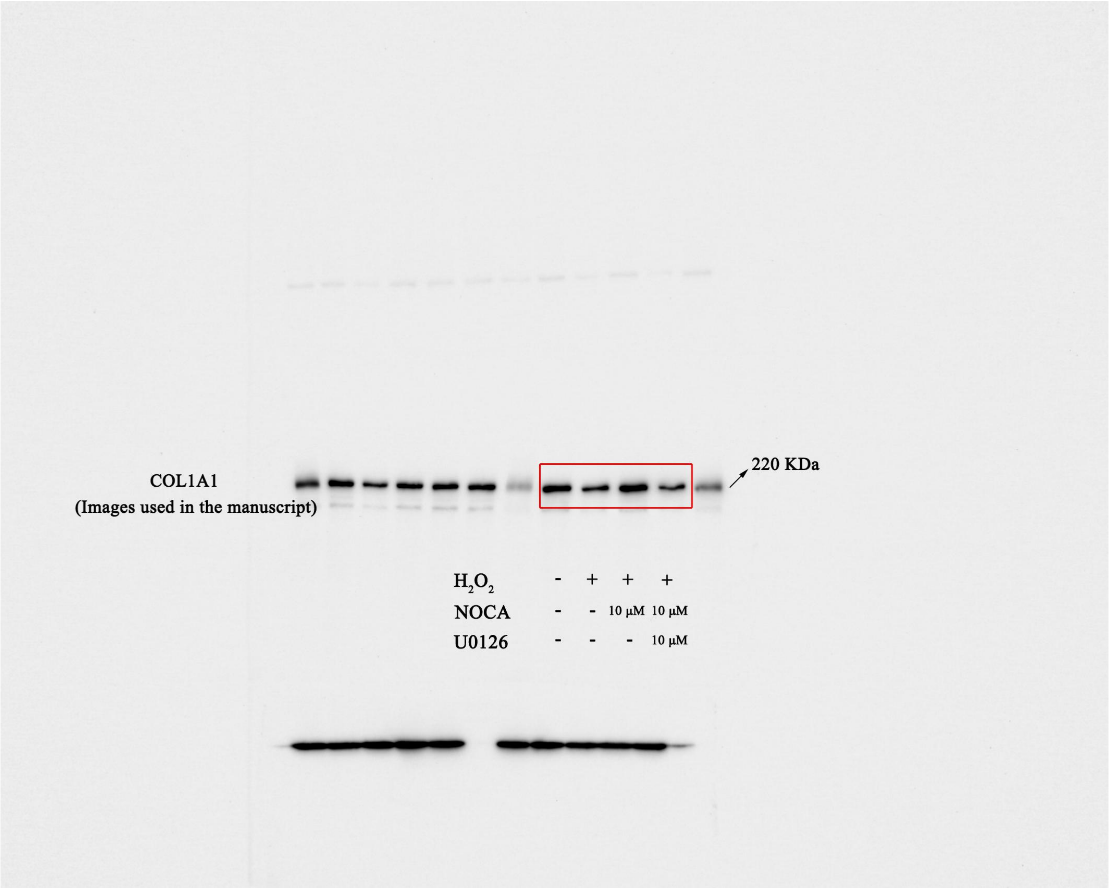

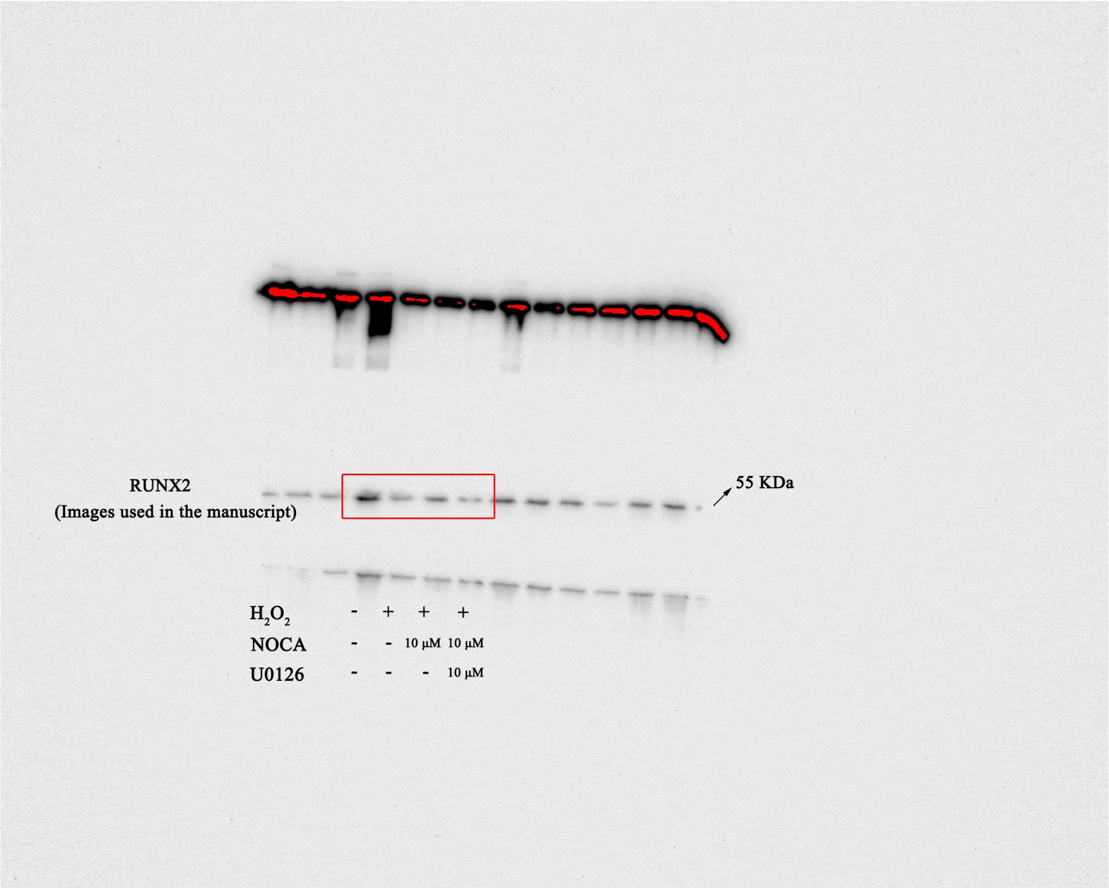

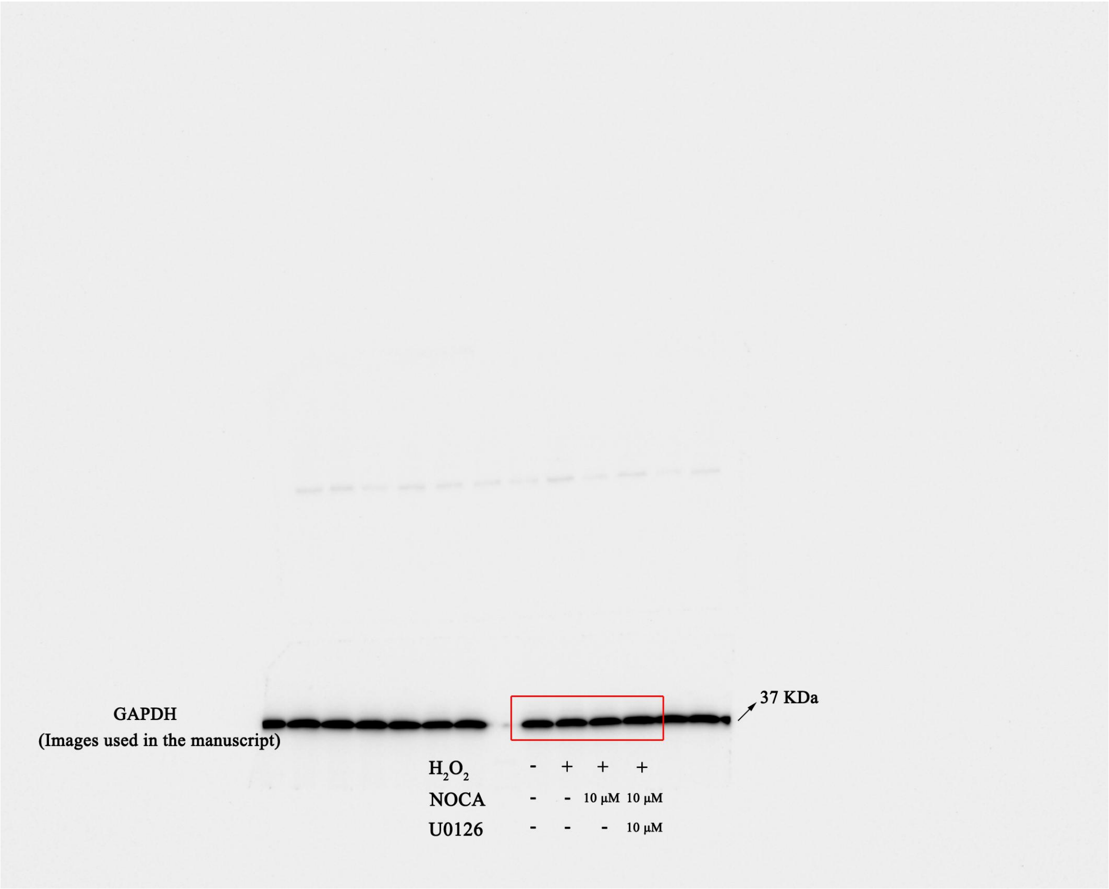
GAPDH
(Images used in the manuscript)
37 KDa
H_2O_2 - + + +
NOCA - - 10 μM 10 μM
U0126 - - - 10 μM

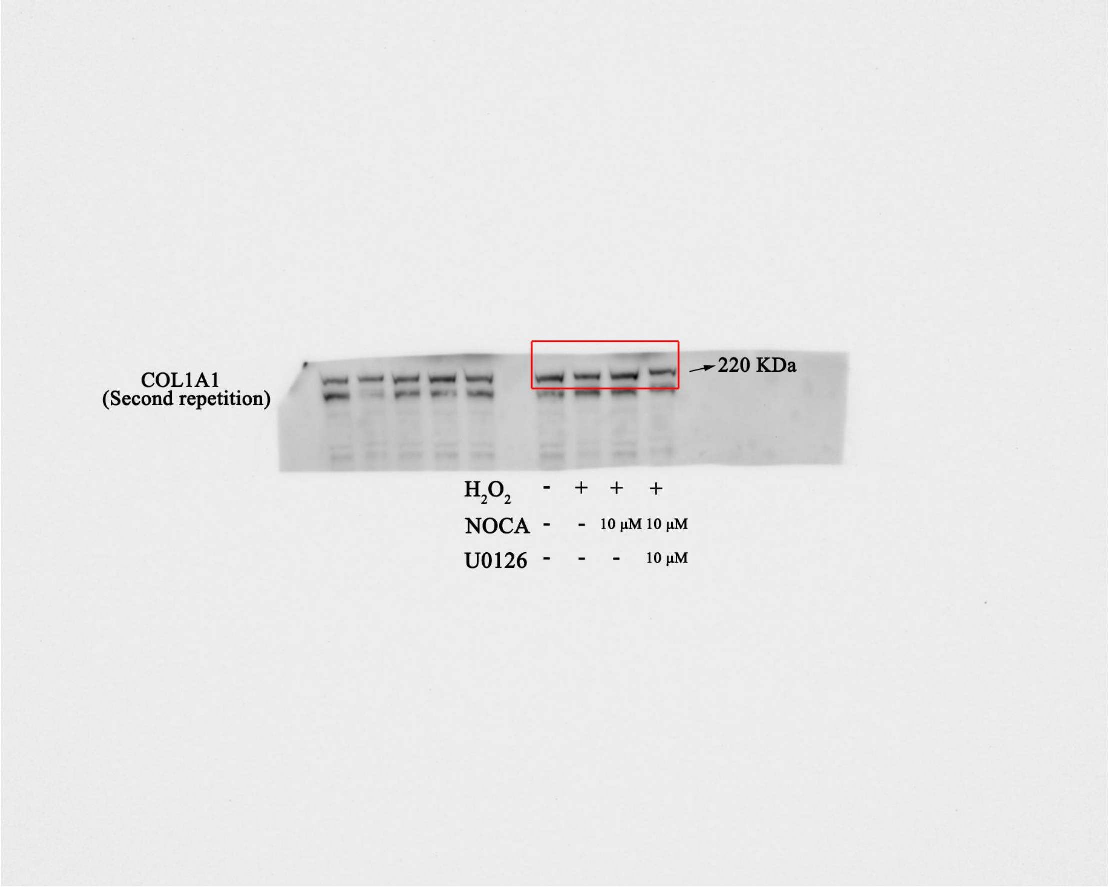
COL1A1
(Second repetition)
220 KDa
H_2O_2 - + + +
NOCA - - 10 μM 10 μM
U0126 - - - 10 μM

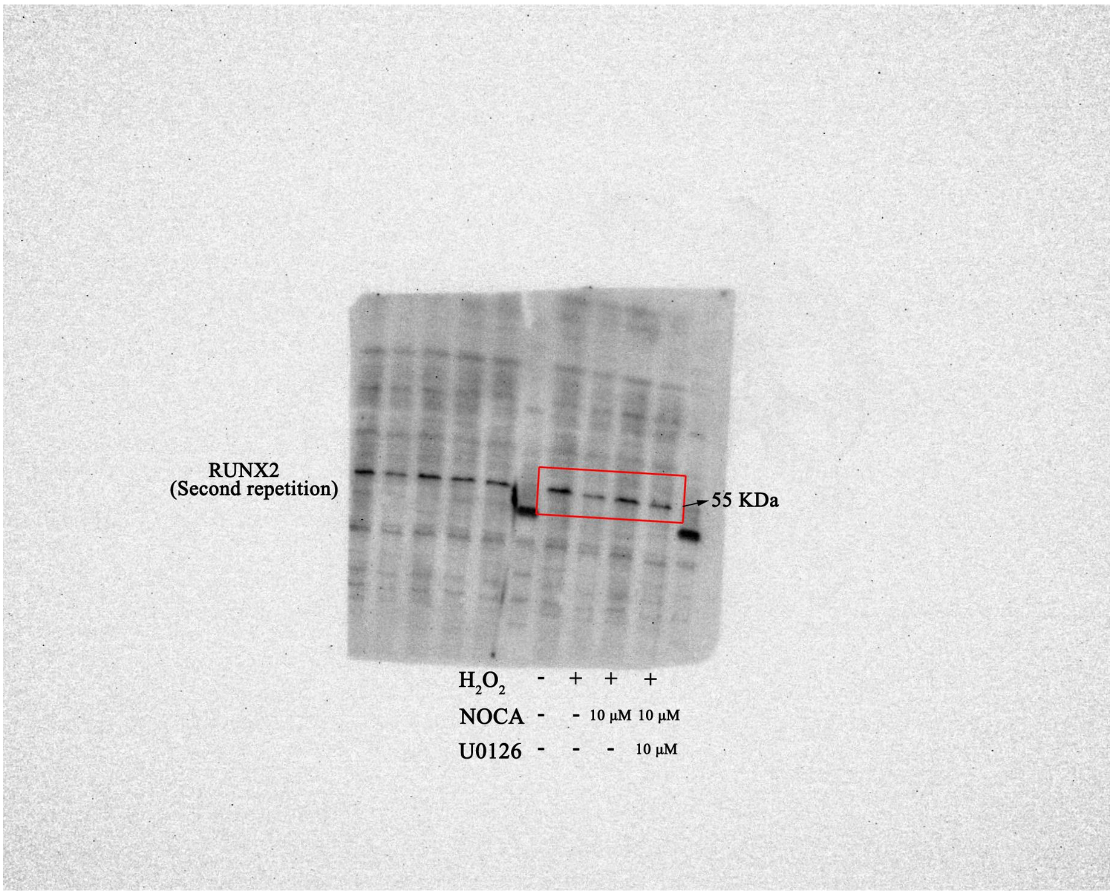
RUNX2
(Second repetition)
55 KDa
H2O2 - + + +
NOCA - - 10 μM 10 μM
U0126 - - - 10 μM

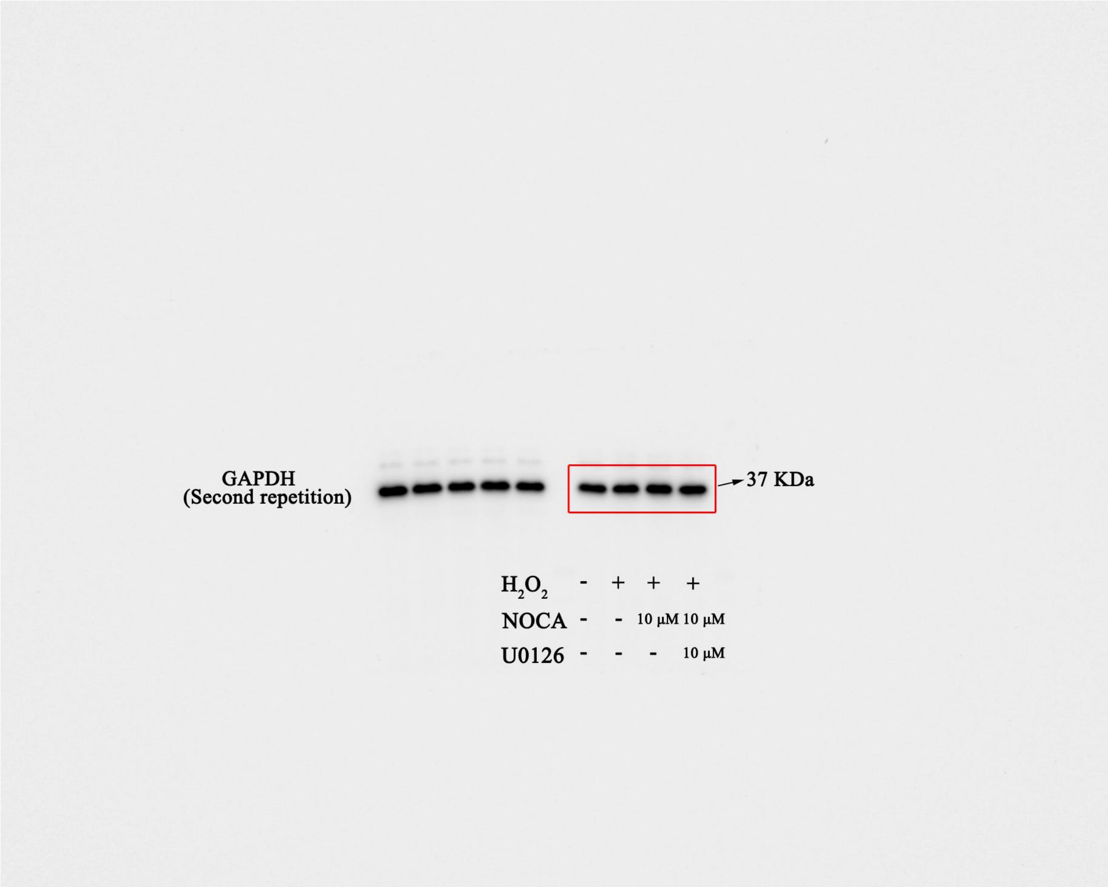
GAPDH
(Second repetition)
37 KDa
H2O2 - + + +
NOCA - - 10 μM 10 μM
U0126 - - - 10 μM

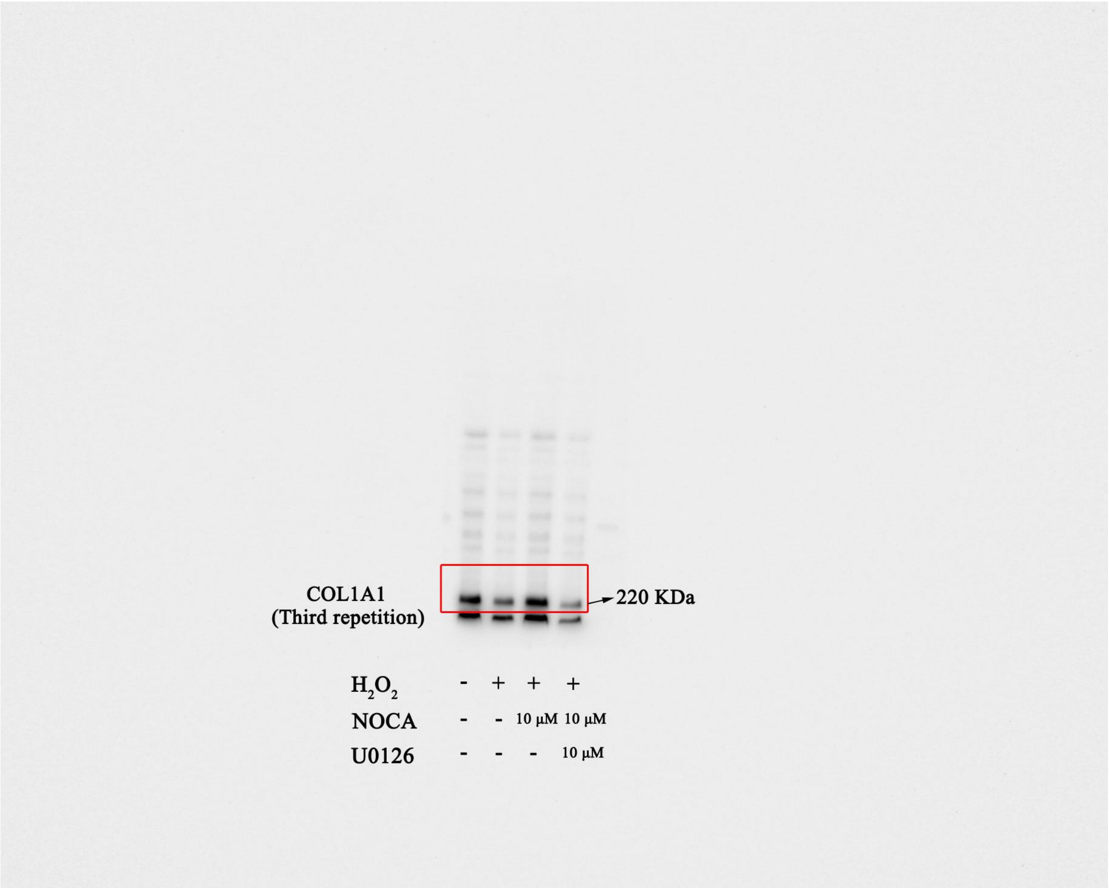
COL1A1
(Third repetition)
220 KDa
H_2O_2 - + + +
NOCA - - 10 μM 10 μM
U0126 - - - 10 μM

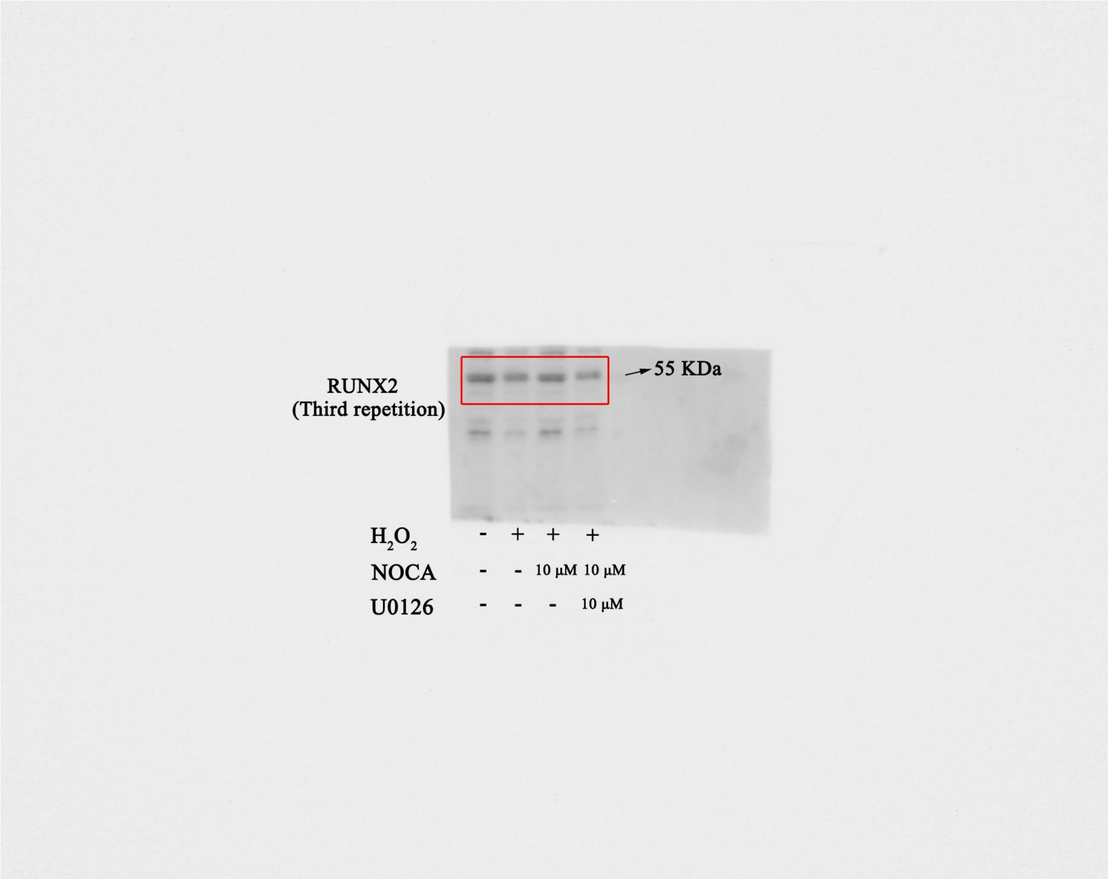
RUNX2
(Third repetition)
55 KDa
H_2O_2 - + + +
NOCA - - 10 μM 10 μM
U0126 - - - 10 μM

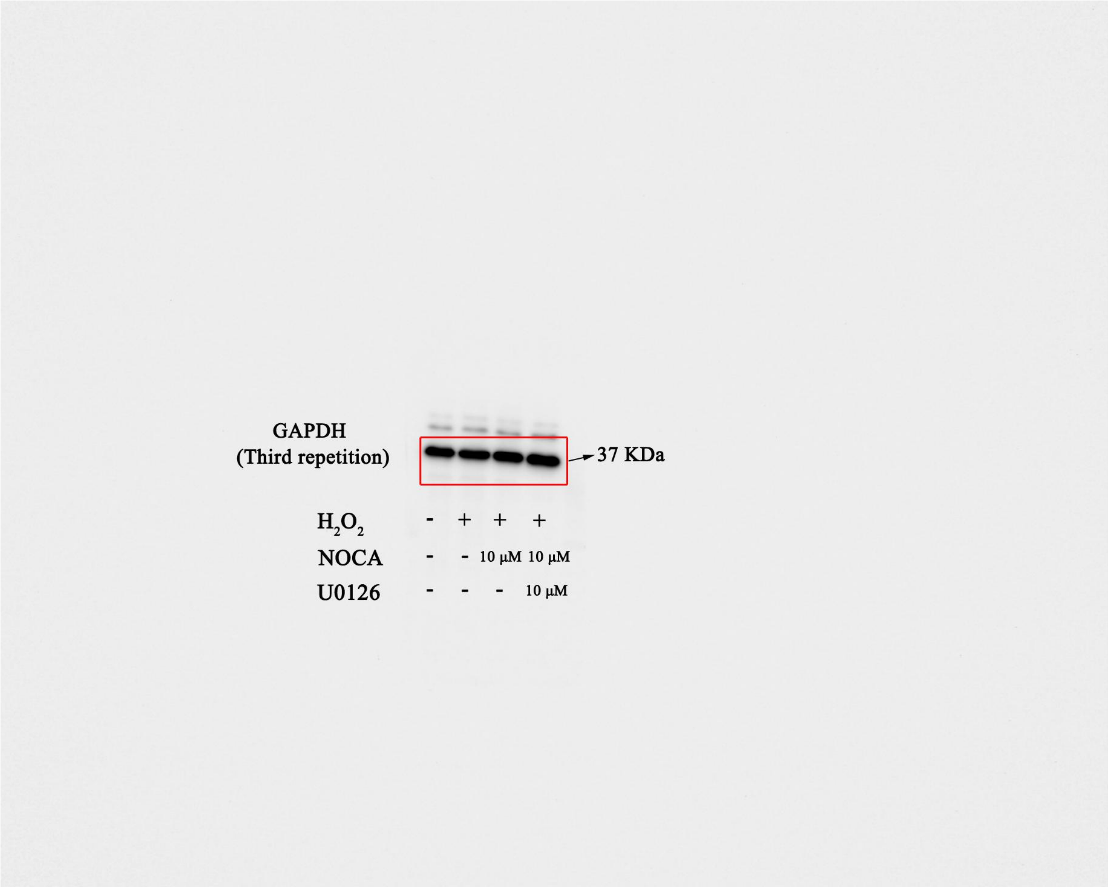

(E) Associated with Figure 7A, D, F

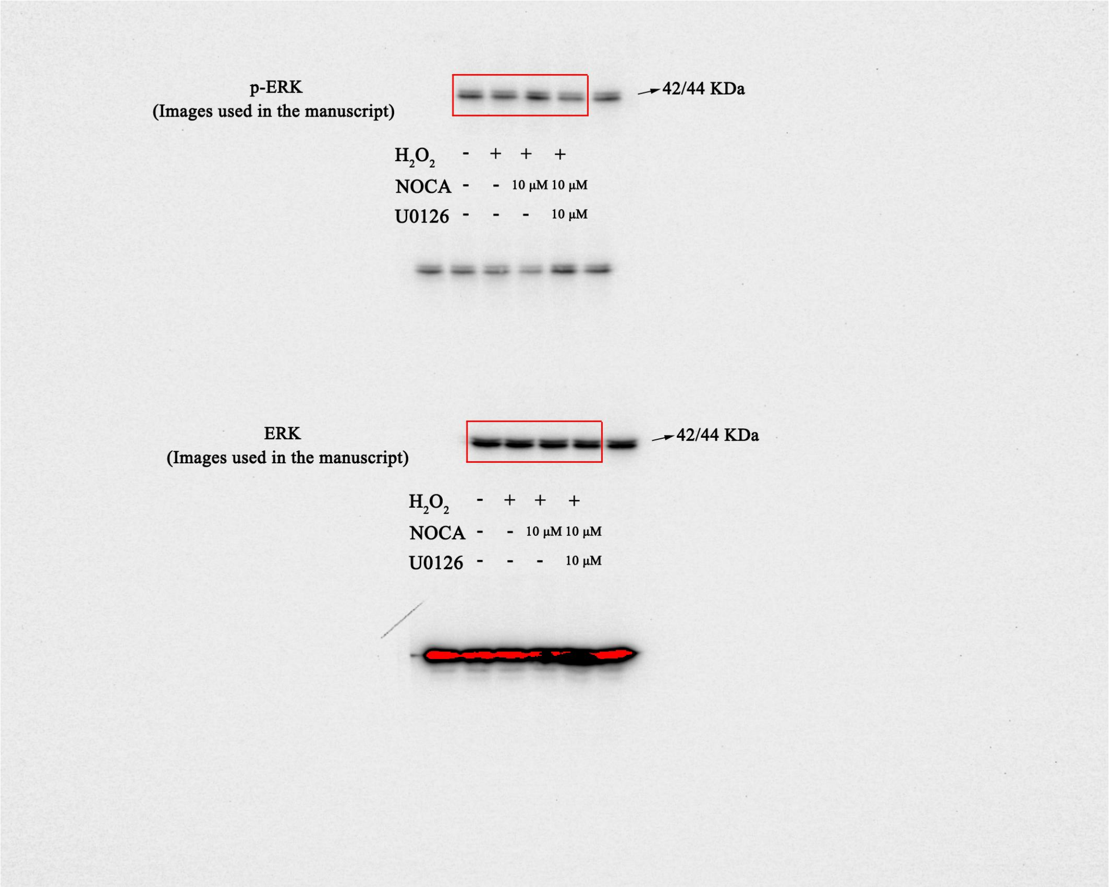
p-ERK
(Images used in the manuscript)
42/44 KDa
H2O2 - + + +
NOCA - - 10 μM 10 μM
U0126 - - - 10 μM
ERK
(Images used in the manuscript)
42/44 KDa
H2O2 - + + +
NOCA - - 10 μM 10 μM
U0126 - - - 10 μM

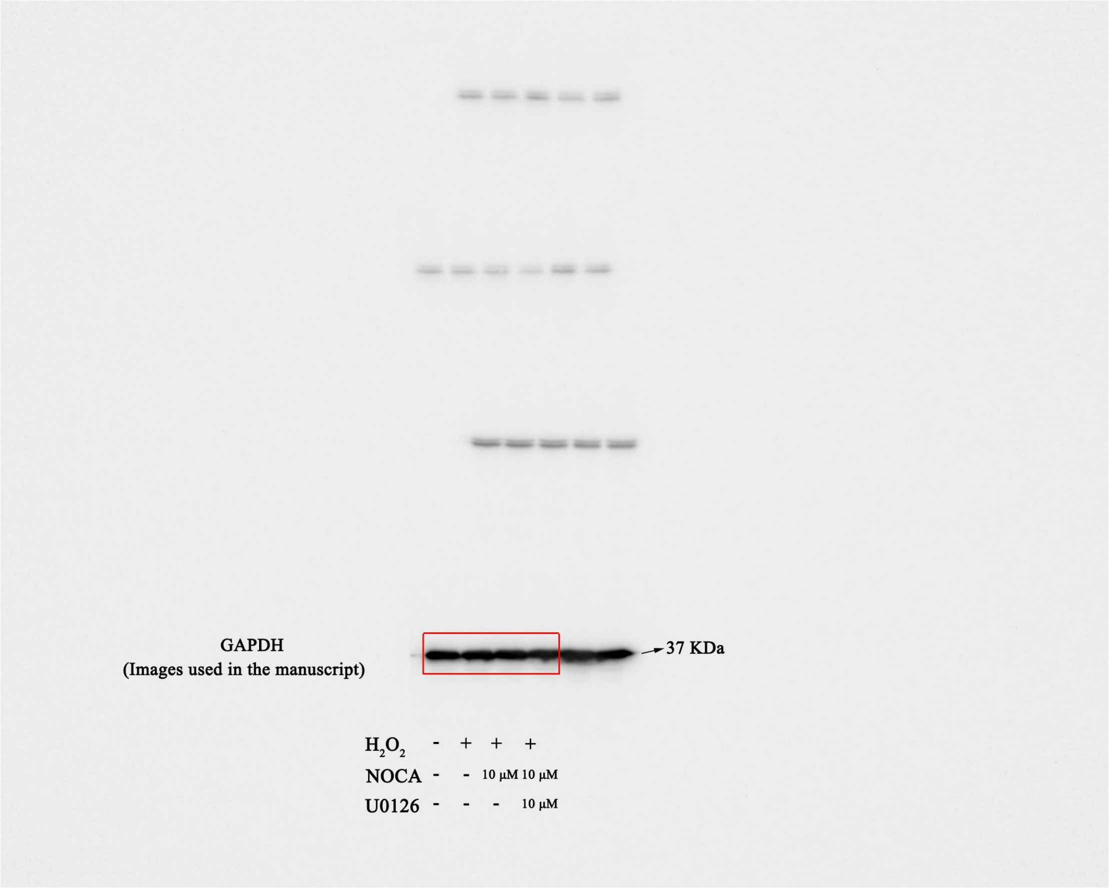
GAPDH
(Images used in the manuscript)
37 KDa
H2O2 - + + +
NOCA - - 10 μM 10 μM
U0126 - - - 10 μM

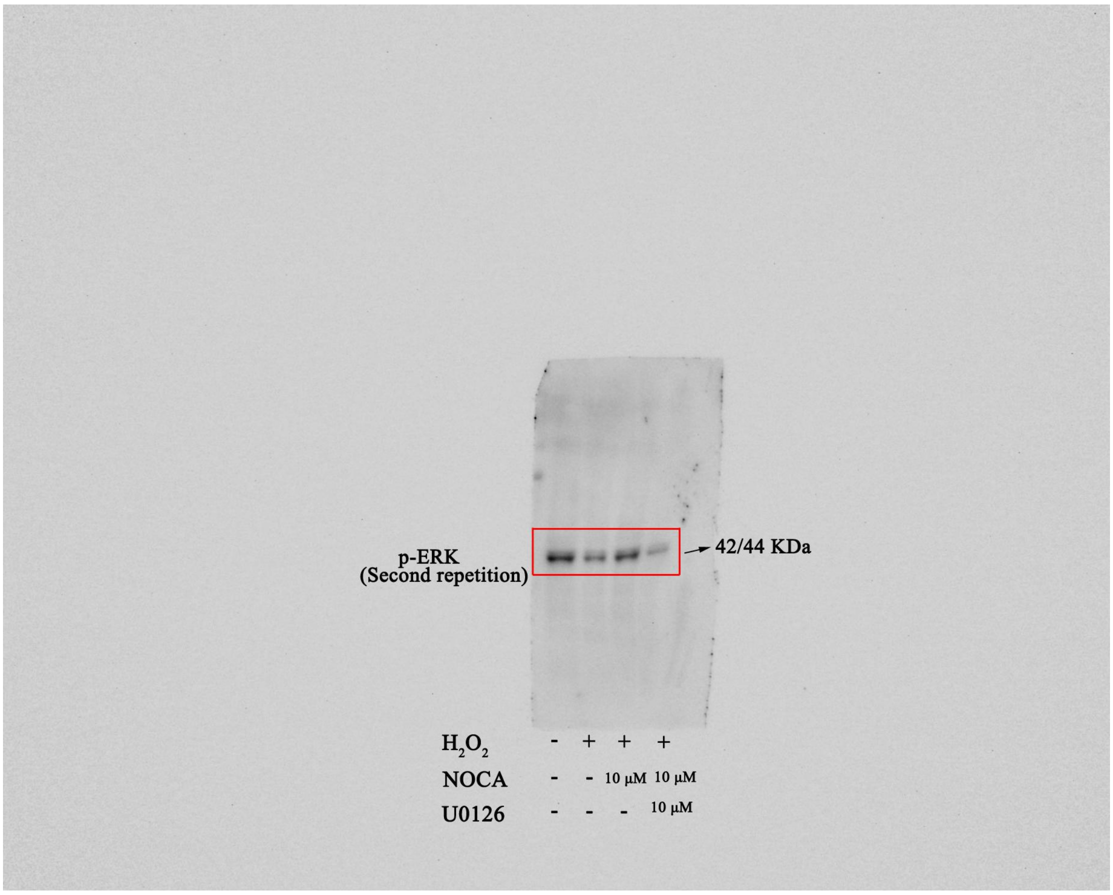
p-ERK
(Second repetition)
42/44 KDa
H2O2 - + + +
NOCA - - 10 μM 10 μM
U0126 - - - 10 μM

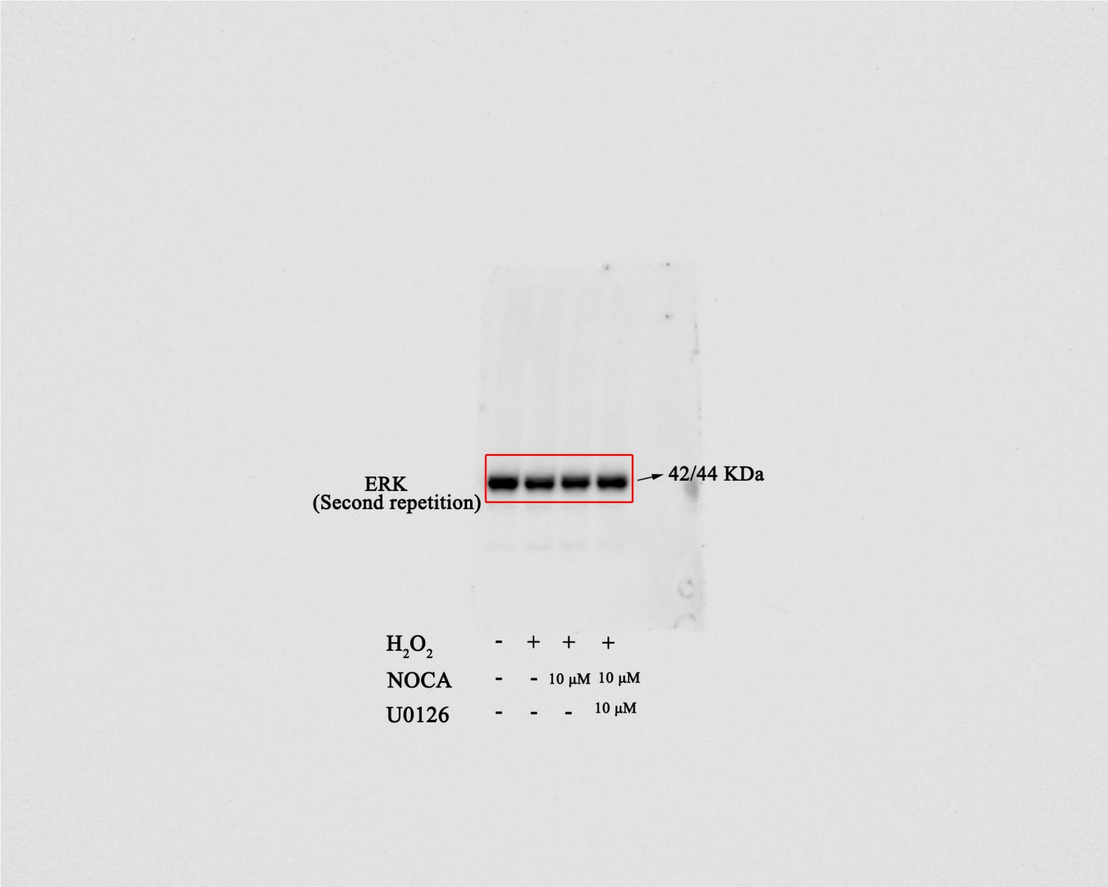
ERK
(Second repetition)
42/44 KDa
H2O2 - + + +
NOCA - - 10 μM 10 μM
U0126 - - - 10 μM

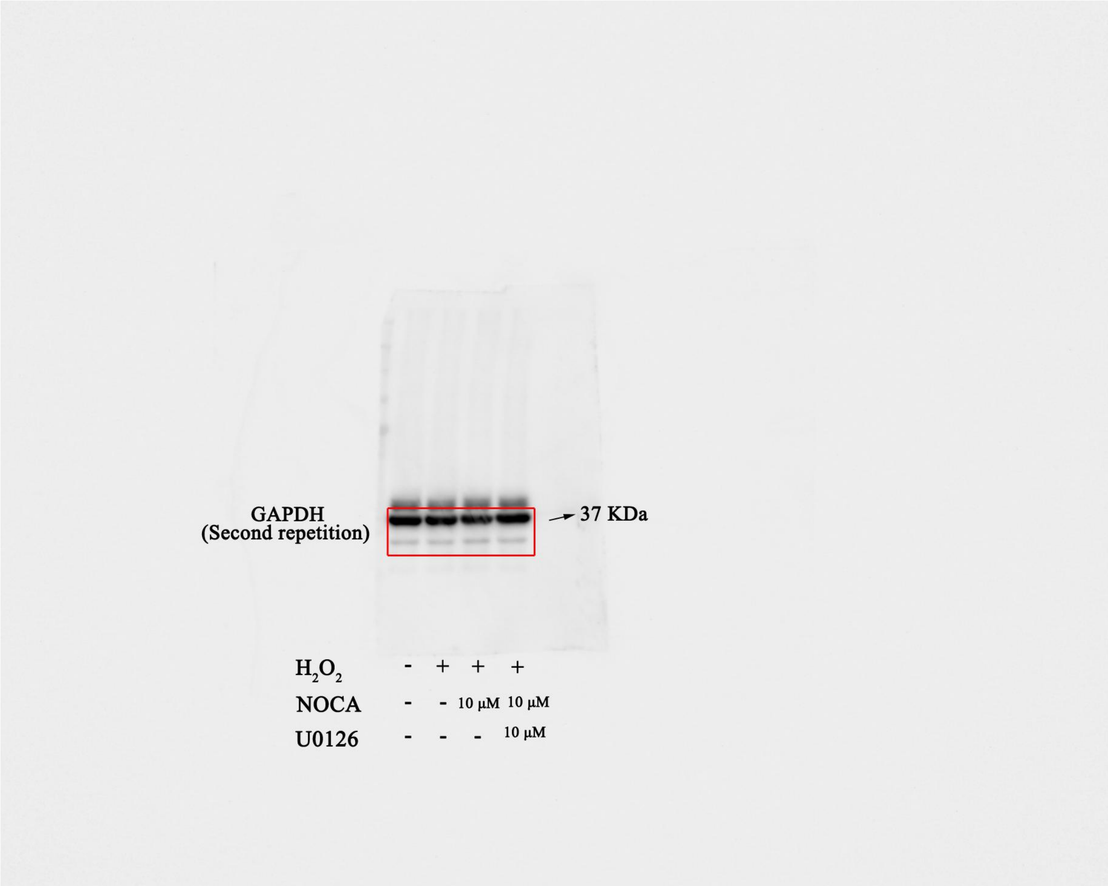
GAPDH
(Second repetition)
37 KDa
H_2O_2 - + + +
NOCA - - 10 μM 10 μM
U0126 - - - 10 μM

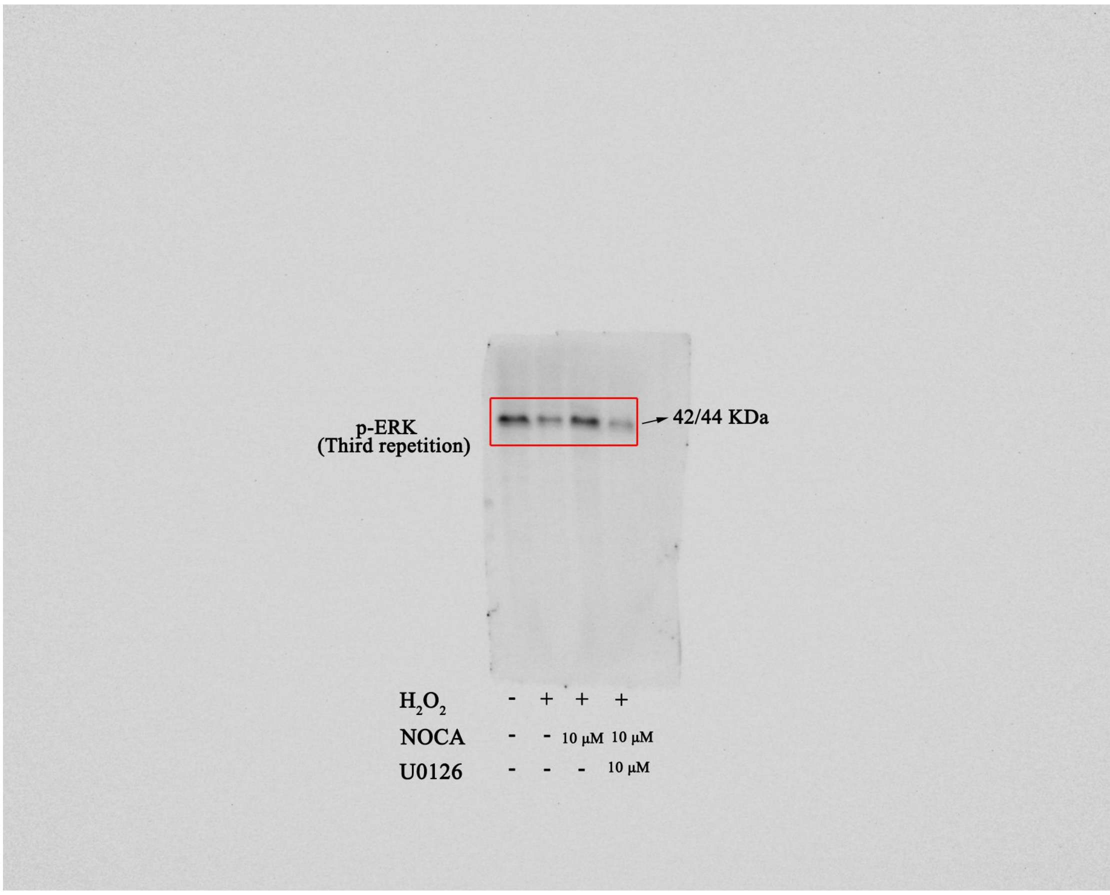
p-ERK
(Third repetition)
42/44 KDa
H_2O_2 - + + +
NOCA - - 10 μM 10 μM
U0126 - - - 10 μM

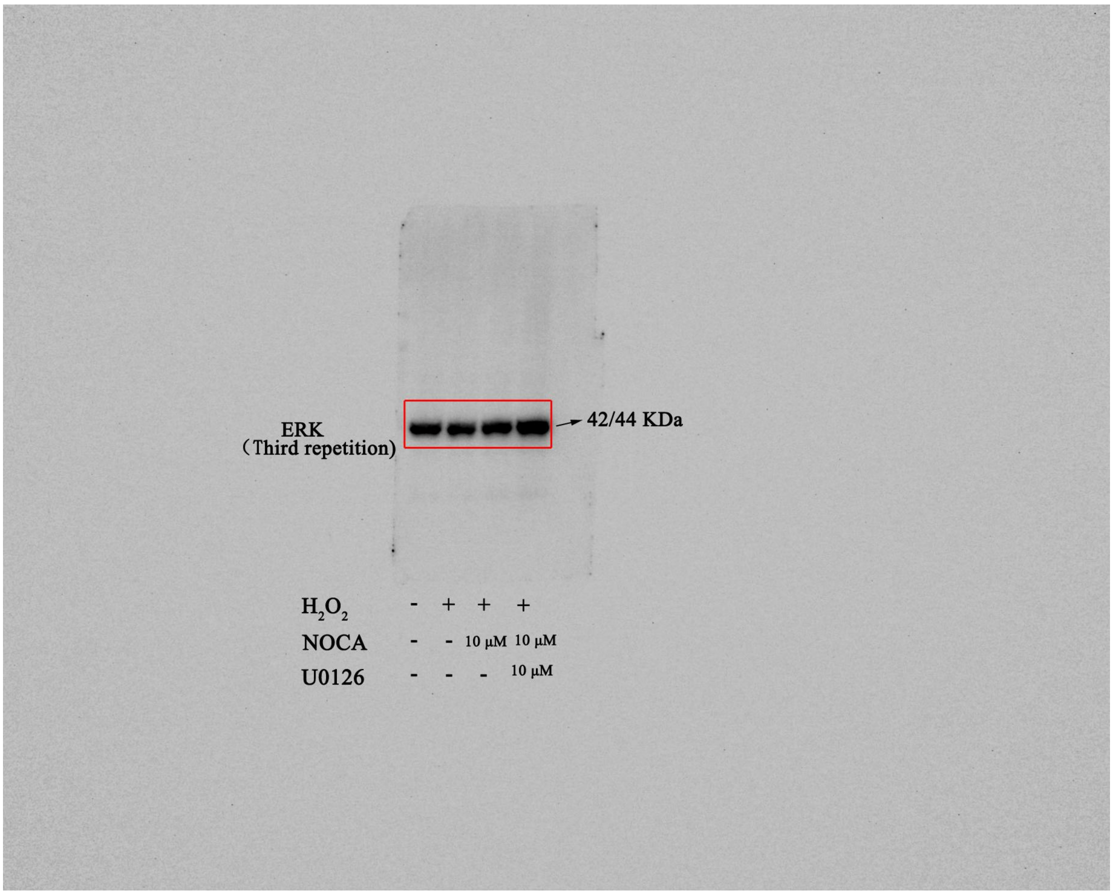
ERK
(Third repetition)
42/44 KDa
H2O2 - + + +
NOCA - - 10 μM 10 μM
U0126 - - - 10 μM

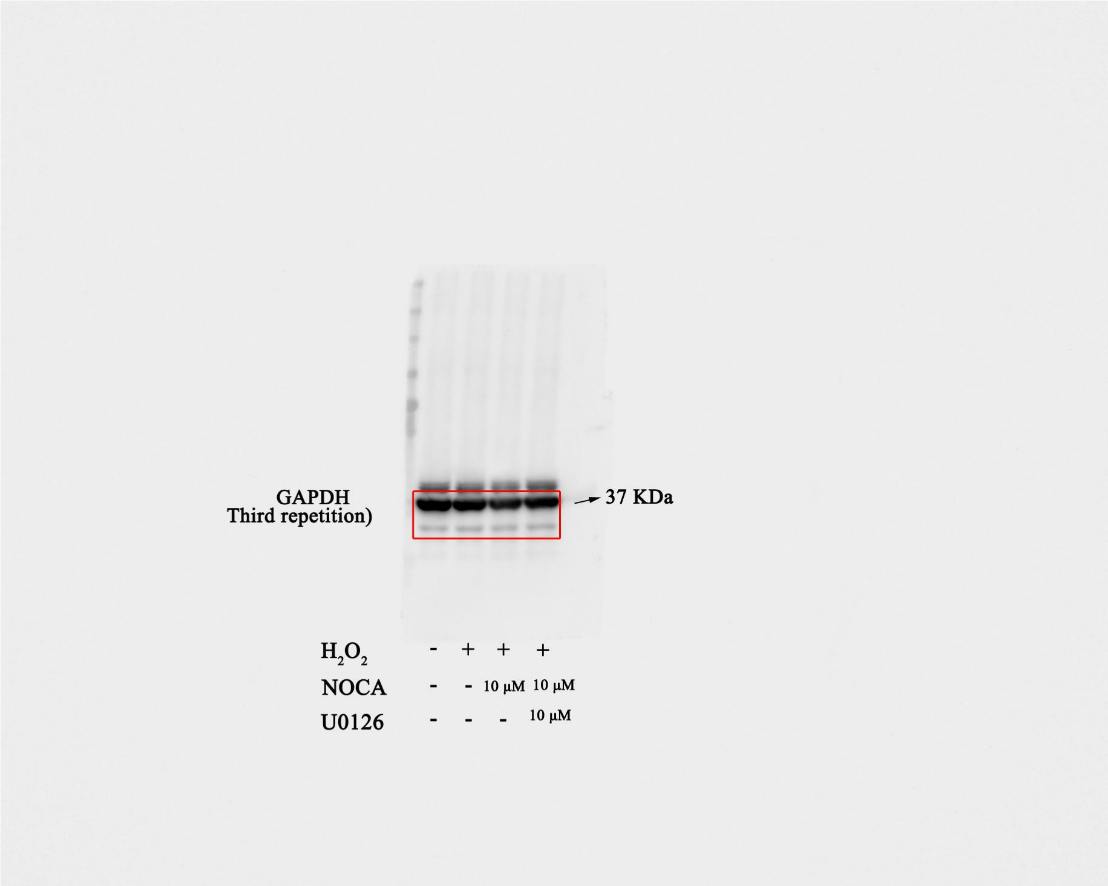
GAPDH
Third repetition)
37 KDa
H2O2 - + + +
NOCA - - 10 μM 10 μM
U0126 - - - 10 μM

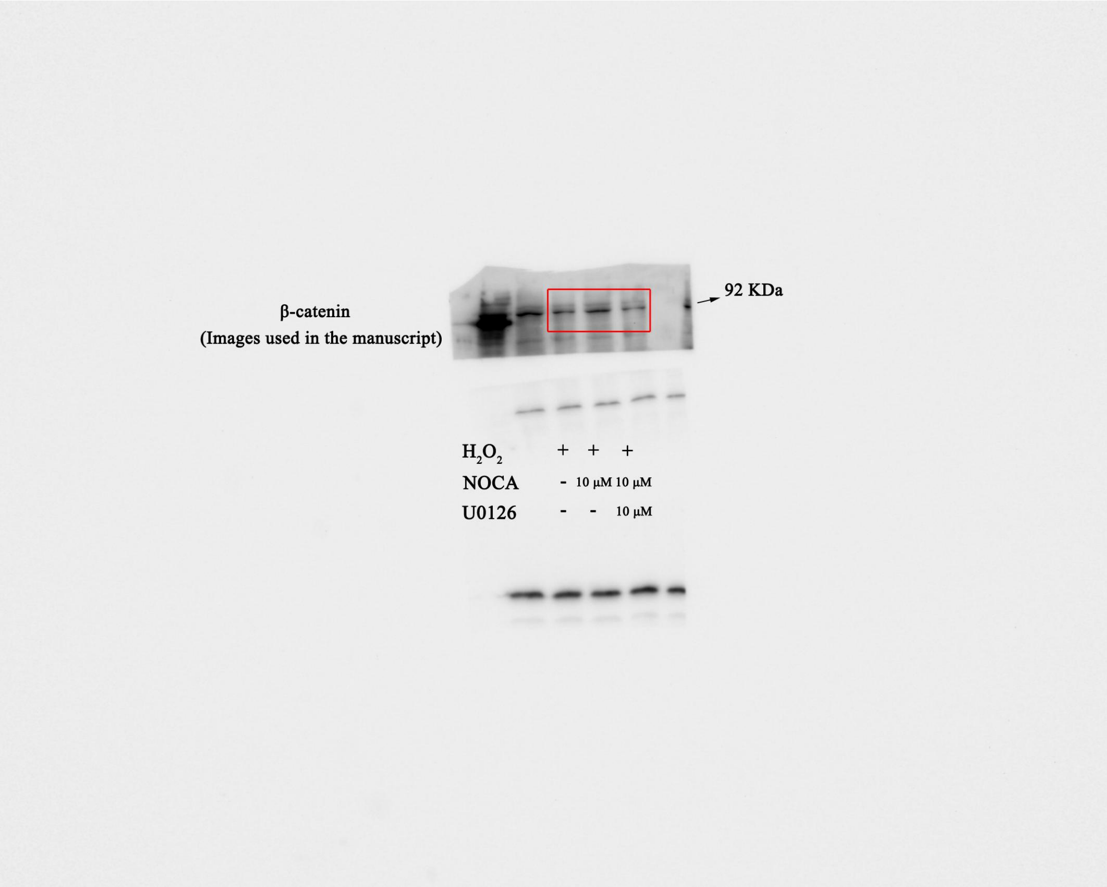
β-catenin
(Images used in the manuscript)
92 KDa
H2O2 + + +
NOCA - 10 μM 10 μM
U0126 - - 10 μM

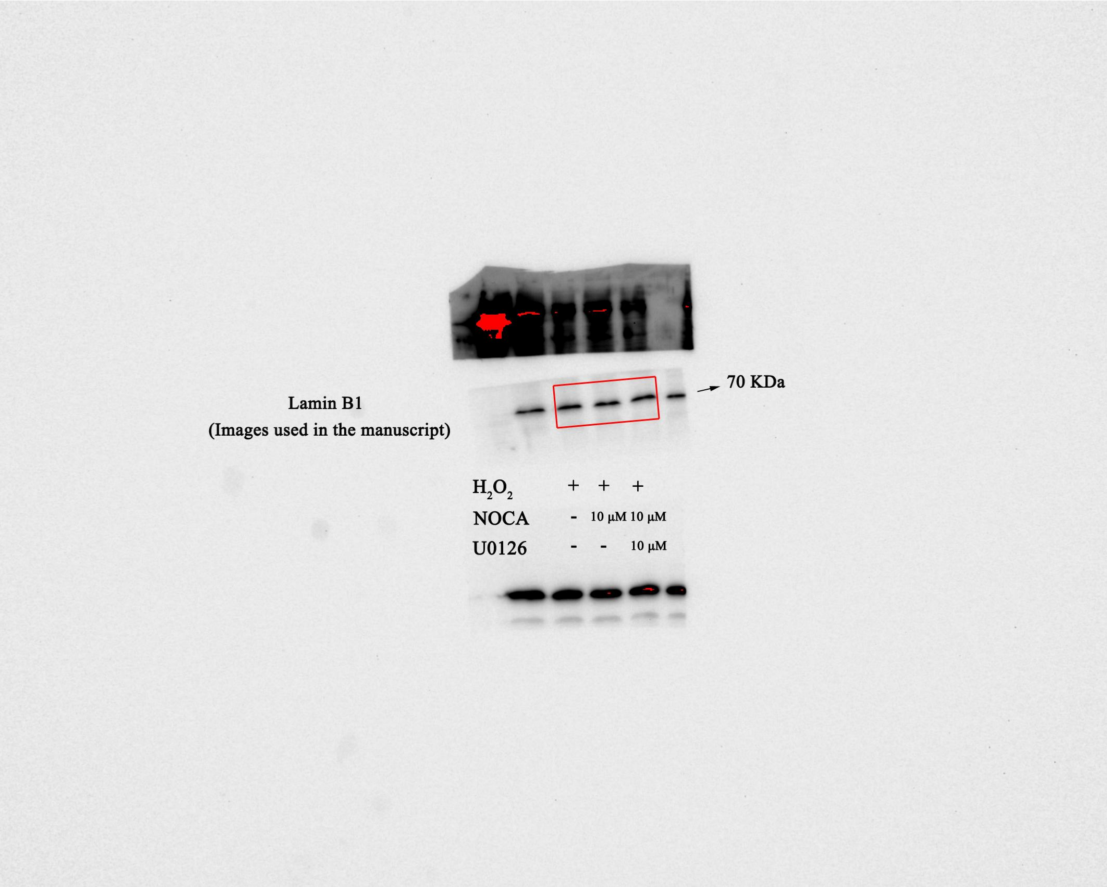
Lamin B1
(Images used in the manuscript)
70 KDa
H2O2 + + +
NOCA - 10 μM 10 μM
U0126 - - 10 μM

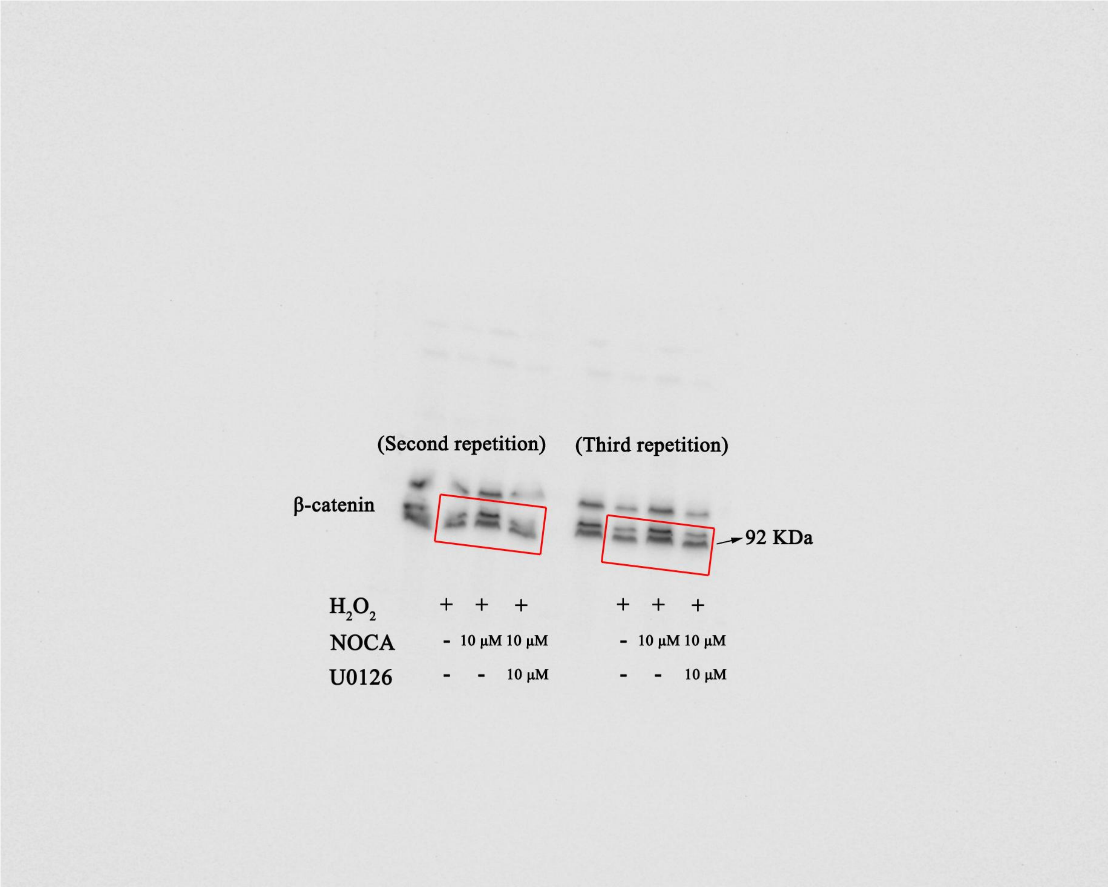
(Second repetition)
(Third repetition)
β-catenin
92 KDa
H_2O_2 + + + + + +
NOCA - 10 μM 10 μM - 10 μM 10 μM
U0126 - - 10 μM - - 10 μM

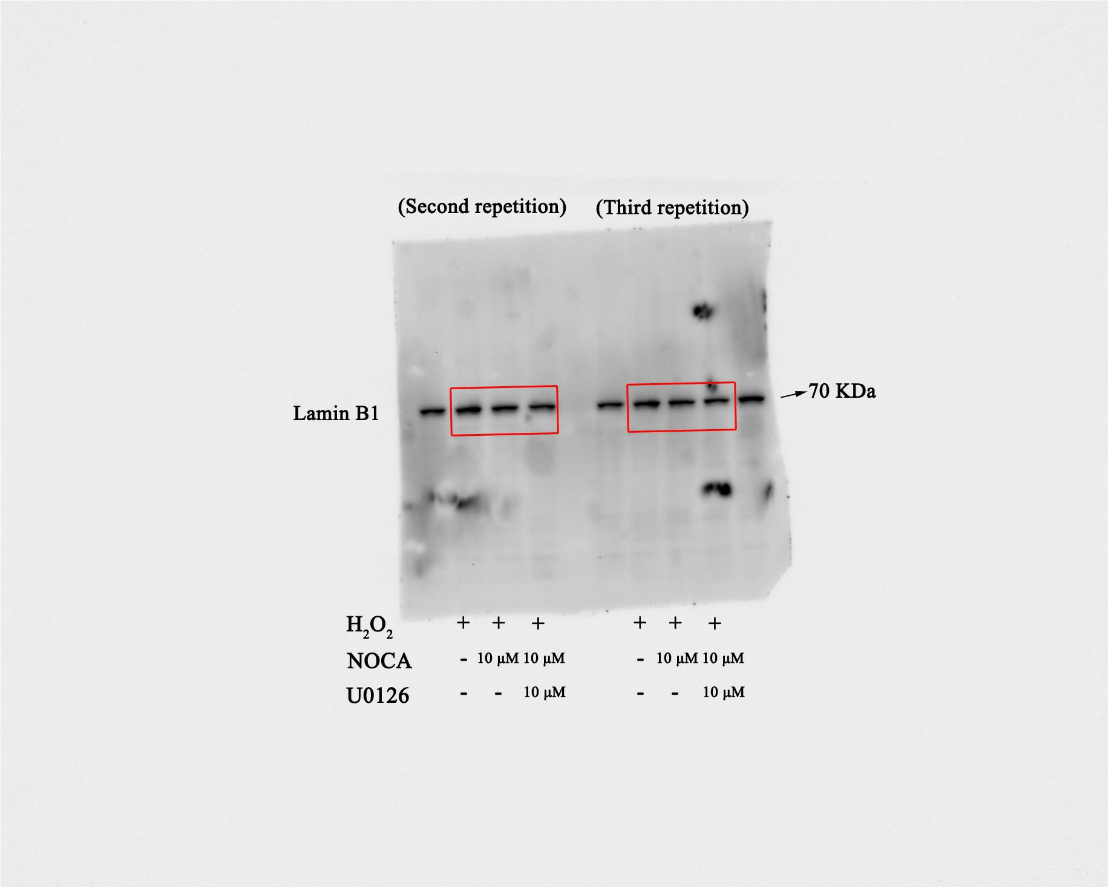
(Second repetition)
(Third repetition)
Lamin B1
70 KDa
H_2O_2 + + + + + +
NOCA - 10 μM 10 μM - 10 μM 10 μM
U0126 - - 10 μM - - 10 μM

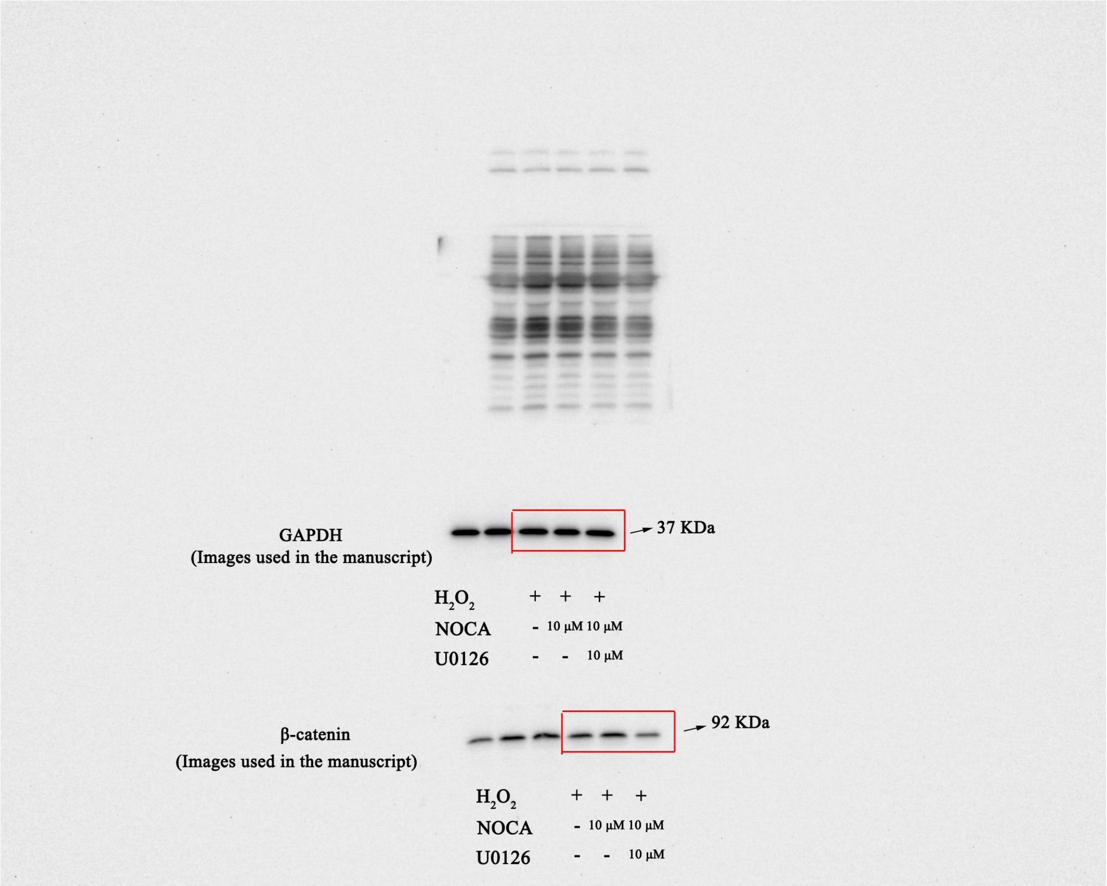
GAPDH
(Images used in the manuscript)
37 KDa
H2O2 + + +
NOCA - 10 μM 10 μM
U0126 - - 10 μM
β-catenin
(Images used in the manuscript)
92 KDa
H2O2 + + +
NOCA - 10 μM 10 μM
U0126 - - 10 μM

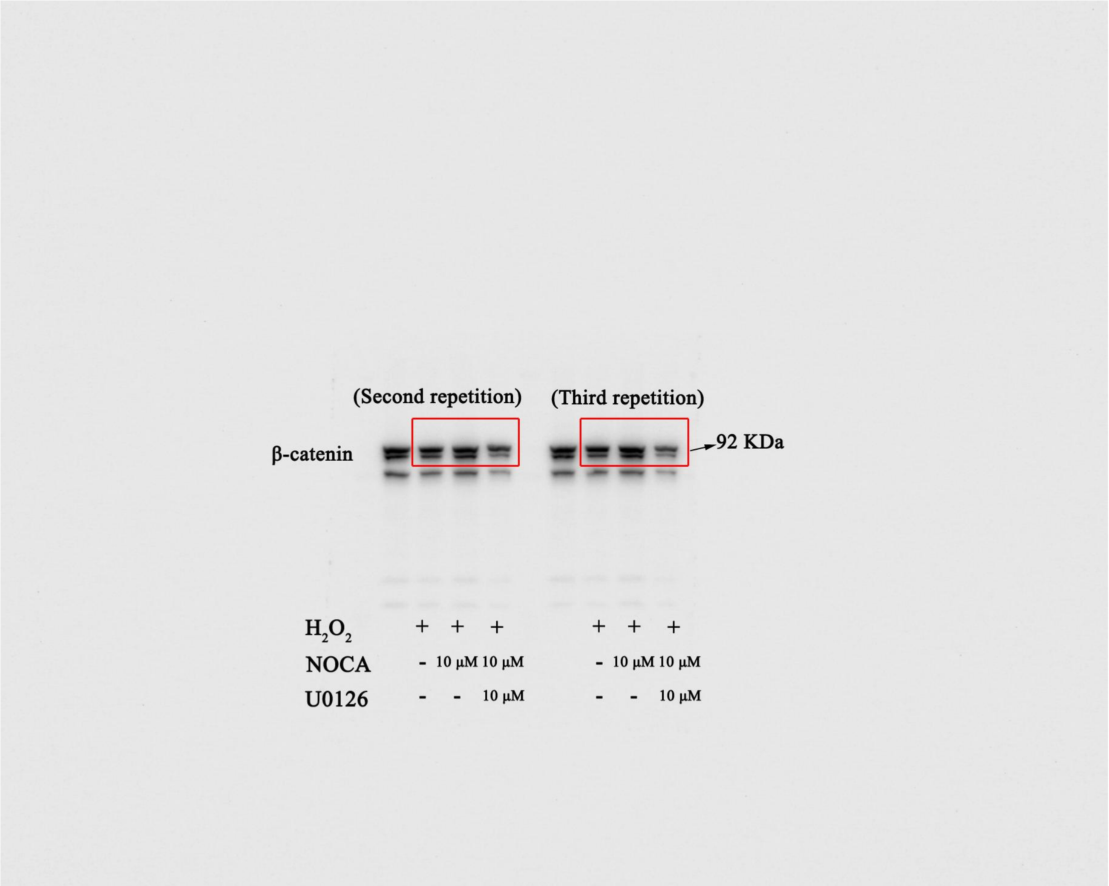
(Second repetition)
(Third repetition)
β-catenin
92 KDa
H2O2 + + + + + +
NOCA - 10 μM 10 μM - 10 μM 10 μM
U0126 - - 10 μM - - 10 μM

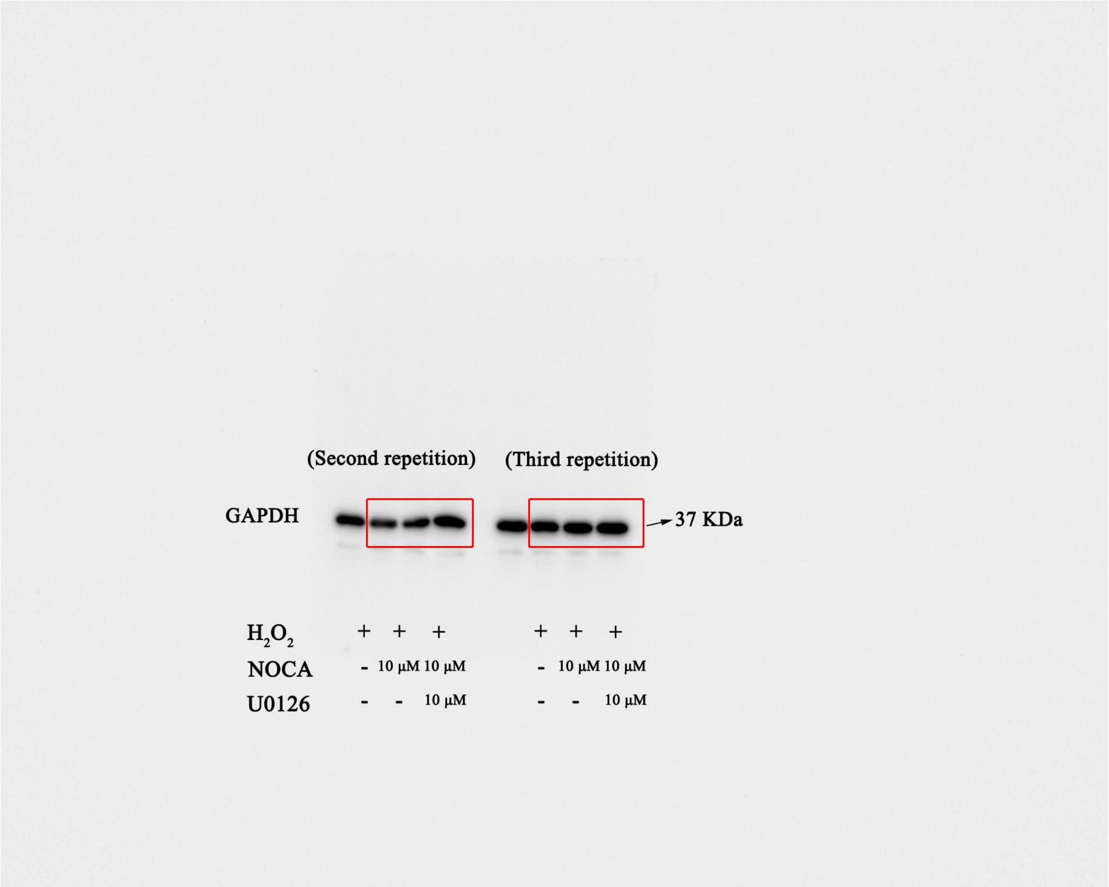
(Second repetition)
(Third repetition)
GAPDH
37 KDa
H2O2
NOCA
U0126
+ + +
+ + +
- 10 μM 10 μM
- 10 μM 10 μM
- - 10 μM
- - 10 μM
